# Genome-Wide Association Study of Grain Appearance and Milling Quality in a Worldwide Collection of *Indica* Rice Germplasm

**DOI:** 10.1371/journal.pone.0145577

**Published:** 2015-12-29

**Authors:** Xianjin Qiu, Yunlong Pang, Zhihua Yuan, Danying Xing, Jianlong Xu, Michael Dingkuhn, Zhikang Li, Guoyou Ye

**Affiliations:** 1 Engineering Research Center of Ecology and Agricultural Use of Wetland, Ministry of Education/College of Agriculture, Yangtze University, Jingzhou 434025, China; 2 Institute of Crop Science/National Key Facility for Crop Gene Resources and Genetic Improvement, Chinese Academy of Agricultural Sciences, Beijing 100081, China; 3 Shenzhen Institute of Breeding & Innovation, Chinese Academy of Agricultural Sciences, Shenzhen 518120, China; 4 International Rice Research Institute, DAPO Box 7777, Metro Manila, Philippines; 5 CIRAD, UMR AGAP, F-34398 Montpellier, France; Aberystwyth University, UNITED KINGDOM

## Abstract

Grain appearance quality and milling quality are the main determinants of market value of rice. Breeding for improved grain quality is a major objective of rice breeding worldwide. Identification of genes/QTL controlling quality traits is the prerequisite for increasing breeding efficiency through marker-assisted selection. Here, we reported a genome-wide association study in *indica* rice to identify QTL associated with 10 appearance and milling quality related traits, including grain length, grain width, grain length to width ratio, grain thickness, thousand grain weight, degree of endosperm chalkiness, percentage of grains with chalkiness, brown rice rate, milled rice rate and head milled rice rate. A diversity panel consisting of 272 *indica* accessions collected worldwide was evaluated in four locations including Hangzhou, Jingzhou, Sanya and Shenzhen representing *indica* rice production environments in China and genotyped using genotyping-by-sequencing and Diversity Arrays Technology based on next-generation sequencing technique called DArTseq™. A wide range of variation was observed for all traits in all environments. A total of 16 different association analysis models were compared to determine the best model for each trait-environment combination. Association mapping based on 18,824 high quality markers yielded 38 QTL for the 10 traits. Five of the detected QTL corresponded to known genes or fine mapped QTL. Among the 33 novel QTL identified, *qDEC1*.*1* (*qGLWR1*.*1*), *qBRR2*.*2* (*qGL2*.*1*), *qTGW2*.*1* (*qGL2*.*2*), *qGW11*.*1* (*qMRR11*.*1*) and *qGL7*.*1* affected multiple traits with relatively large effects and/or were detected in multiple environments. The research provided an insight of the genetic architecture of rice grain quality and important information for mining genes/QTL with large effects within *indica* accessions for rice breeding.

## Introduction

Rice (*Oryza sativa* L.), a staple cereal crop, feeds more than half of the world’s population. Improvement of rice yield and grain quality is the major objective of rice breeding worldwide. Grain quality primarily includes grain appearance, milling, eating and cooking and nutrition qualities. Grain appearance quality mainly includes grain shape and chalkiness. Grain shape, described by grain length (GL), grain width (GW), grain thickness (GT) and grain length to width ratio (GLWR), contributes to grain weight and yield and has a great impact on the market values of rice grain products. A short and round rice grain is generally preferred by consumers in Northern China, Korea and Japan, whereas consumers in Southern China, the USA, and South and Southeast Asian countries prefer a long and slender rice grain [1]. Chalkiness, according to its location within the endosperm, can be divided into white belly, white core and white back in rice grain [2]. Chalky grains, filled with loosely packed, round and large compound of starch granules, are more prone to breakage during milling and reduce head milled rice rate (HMRR). Furthermore, when chalky grains are cooked, cracks occur easily and reduce the palatability of the cooked rice [3]. Degree of endosperm chalkiness (DEC) and percentage of grains with chalkiness (PGWC) are commonly used to measure grain chalkiness. Milling quality determines the final yield and the broken kernel rate of the milled rice, which is of concern for consumers and farmers. Milling quality is measured by brown rice rate (BRR), milled rice rate (MRR) and HMRR. Brown rice is the de-hulled rice with the palea and lemma removed that can be used for cooking and eating. Milled rice is the result of brown rice after removing all of the bran, which consists of aleurone and pericarp, and germ or embryo. Head milled rice is kernel longer than or equal to 3/4 full length of a kernel. Among the above-mentioned three milling quality parameters, HMRR is the most important and greatly affects market value. HMRR depends on varietal characteristics, production factors, and harvesting, drying and milling processes.

Most of the rice quality determining traits are quantitatively inherited, controlled by multiple genes/QTL [2] and affected by growing environment [4]. As for other complex traits, QTL mapping using bi-parental populations derived from parental lines of contrast performance has been widely used to identify QTL for various rice quality traits. Many genes/QTL for rice grain appearance quality and milling quality traits have been reported in the last decades. The *GW1-1* and *GW1-2* [5], *qGRL1*.*1* [6], *GS2* [7], *GW3* and *GW6* [8], *qGL-4b* [9], *qPGWC-7* [10] *qGL-7* [11], *qGRL7*.*1* [6], *gw8*.*1* [12], *gw9*.*1* [13], *tgw11* [14] have been fine mapped. The *GW2* [15], *GS3* [16], *qGL-3* [17], *qSW5* [18], *GS5* [19], *Chalk5* [20], *TGW6* [21], *GW6a* [22], *SRS1* [23], *GL7*/*GW7* [24, 25], *GW8* [26] and *CycT1;3* [27] have been cloned. The usefulness of some of the well characterized genes/QTL was proven in an *indica* population of diverse breeding lines [4].

However, QTL mapping using a single bi-parental population has a few important limitations including the need to create a population segregating for the target trait, the ability to assess only two alleles per locus, and the limited number of meiosis. Only a limited number of QTL can be identified, since only QTL for which the two parents differ can be detected. Given the limited scope of each study, mapping the same trait in different bi-parental populations may yield different QTL. More than two alleles are likely to segregate per locus, and the directions of QTL effects may vary depending on the genetic background because of epistasis, pleiotropy and QTL- by- environment interaction (QEI) [28].

An alternative QTL mapping approach is to conduct association analysis in a large germplasm collection, known as association mapping. Association mapping is based on linkage disequilibrium (LD) or the non-independence of alleles in a population. Association mapping studies using sparse SSR markers has been proven to be successful in identifying marker-trait associations (MTA) in rice [29–32]. With the development of high-throughput sequencing and SNP chip techniques, genome-wide association study (GWAS) using high density markers becomes more and more popular in rice genetics. The first GWAS in rice was conducted by Huang et al. [33] using 517 rice landraces genotyped by sallow sequencing. Among the QTL identified two and five were for GW and GL, respectively [33]. Applying the same method to a large panel of 950 worldwide rice accessions, Huang et al. [34] identified two, four and two QTL for GW, GL and thousand grain weight (TGW), respectively. Based on genotyping 44,100 SNP across 413 diverse accessions from 82 countries, Zhao et al. [35] identified eight,10 and 15 QTL for GW, GL and GLWR, respectively. Begum et al. [36] identified seven, 10 and 11 QTL for GW, GL and GLWR, respectively, using advanced lines from IRRI’s irrigated breeding program genotyped with 71,710 SNP generated by Genotyping-by-sequencing (GBS). Similarly, using 533 *O*. *sativa* landrace and elite accessions genotyped with 4,358,600 SNP derived from GBS, Yang et al. [37] identified 10, 11, 19 and 21 QTL for GLWR, TGW, GW and GL, respectively. These studies clearly demonstrated that GWAS offers higher resolution for QTL mapping and can be used as a powerful complementary strategy to the classical linkage mapping using bi-parental segregation populations.

Many of the reported association studies in rice included grain traits, such as GL, GW, GLWR, GT and TGW. However, rice chalkiness and milling quality traits were less studied. The objective of this study is to identify markers associated with 10 grain appearance quality and milling quality traits using an *indica* collection of 272 accessions. The panel was phenotyped in four locations representing major *indica* rice production environments in China. GBS and Diversity Arrays Technology (DArT) based on next-generation sequencing technique called DArTseq™ was used to generate genome-wide markers. The association panel was characterized for population structure using three different methods and for LD pattern by estimating basal LD and LD decay in the whole population and subpopulations. Sixteen association mapping models were tested to choose the best model for each of the trait-environment combinations. A number of known QTL and novel QTL were identified.

## Materials and Methods

### Association mapping panel

A total of 272 *indica* rice accessions from 31 countries or regions (S1 Table) were used in this study. More accessions came from China (39), Philippines (36), Madagascar (29), India (28), Senegal (24), Sri Lanka (24) and Bangladesh (15). For other countries or regions the number of accessions was fewer than eight.

### Phenotypic evaluation

Field trials were conducted using a randomized complete block design with two replicates in four environments including Hangzhou (HZ), Jingzhou (JZ), Sanya (SY) and Shenzhen (SZ) in China. The sowing and transplanting dates varied for the testing environments to fit into the local planting season. In all environments, the plot size was three rows of 10 plants planted at a spacing of 20cm x 25cm. Local farmers’ management practices were followed. At maturity, eight plants in the middle row were harvested. The institutions including Yangtze University, Zhejiang Academy of Agricultural Sciences, Shenzhen Institute of Breeding & Innovation permitted the field trials were conducted in their experimental fields.

The nature dried seeds stored in room temperature for three months were used to measure quality traits. GL (mm), GW (mm), GLWR, GT (mm), TGW (g), BRR (%), MRR (%) and HMRR (%) were measured according to the National Rice Grain Quality Assessment Standard of China (GB/T17891-1999). The grain chalkiness characteristics were measured using a JMWT12 Rice Appearance Quality Detector (Dong Fu Jiu Heng, Beijing). PGWC (%) was the percentage of head milled grains with chalkiness. DEC (%) was calculated as the product of PGWC and chalkiness size (the area of chalkiness divided by the area of whole grain). All measurements were conducted for two independent samples.

### Phenotypic analysis

Due to various reasons not all accessions had phenotypic data in all the four testing environments. The final population size for each of the trait-environment combinations varied greatly (Table 1). Phenotypic analysis was conducted using linear mixed models to properly handle the unbalance data. For single-site analysis, accession (genotype) was regarded as fixed effect and replicate as random effect. The best linear unbiased estimates (BLUE) of accessions were obtained. For multi-site analysis, all effects including accession (genotype), environment and replicate within environment were regarded as random to estimate variance components. The best linear unbiased predictions (BLUP) for each of the genotype-environment combinations were predicted. All analyses were conducted using the PBTools package of R [38] developed by IRRI (bbi.irri.org). Phenotypic correlations were computed from the BLUE using the “rcorr” function implemented in the R package Hmisc [39]. Narrow-sense heritability (h^2^) based on genotypic means was computed using the estimated variance components as V_G_ / (V_G_ + V_GEI_ /s +V_e_ /sr). Where, V_G_, V_GEI_, V_e_ are the variance of genotype, genotype by environment interaction (GEI) and residual error, respectively, s is the number of environments and r is the number of replicates.

**Table 1:** Popoation size for 10 traits in four environments.

Trait	HZ	JZ	SY	SZ
GL	220	131	216	228
GW	218	132	213	229
GLWR	218	131	215	229
GT	198	132	217	221
TGW	167	130	214	206
DEC	147	128	206	197
PGWC	147	131	213	200
BRR	136	130	201	206
MRR	136	130	213	205
HMRR	136	130	213	205

### Genotyping

Genomic DNA was extracted for each accession from the leaf tissues of a single plant using the MATAB method described in Risterucci et al. [40] and then diluted to 100 ng/μl. Genotyping was conducted at Diversity Arrays Technology Pty Ltd. (DArT P/L), Australia, using a method of DArTseq™. The method involves genome complexity reduction using *Pst*I/*Taq*I restriction enzymes followed by Illumina short-read sequencing. *Pst*I specific adapters tagged with 96 different barcodes to encode a plate of DNA samples were ligated to the restriction fragments. The resulting products were amplified and checked for quality. The 96 samples were pooled and run in a single lane on an Illumina Hiseq2000 instrument. The *Pst*I adapters included a sequencing primer so that the tags generated were always read from the *Pst*I sites. As detailed by Courtois et al. [41], the resulting sequences were filtered and split into their respective target datasets, and the barcode sequences were trimmed. The sequences were trimmed at 69 bp (5 bp of the restriction site plus 64 bases with a minimum Q score of 10). A proprietary analytical pipeline developed by DArT P/L was used to produce DArT score tables and SNP tables. The remaining 69 bp sequences were aligned to the Os-Nipponbare-Reference-IRGSP-1.0 [42] pseudomolecule assembly using Bowtie v0.12 [43] with a maximum of three mismatches to recover the position of the restriction site for the DArT markers and the position of the polymorphism(s) within the 69 bp sequences for the SNPs. For the DArT markers, the position given is that of the second base of the 6 base *Pst*I restriction site (5'-C|TGCAG-3') because the mutated base is unknown and can be any of the six. The same sequences were then aligned to the pseudomolecules using BLAST (e-value <1.0 e-20) to assess whether additional sequences could be positioned. The sequences that had only one hit on the pseudomolecules or had more than one hit but with a difference of at least 1.0 e-5 between the first and the second hits were retained for further analyses. When the marker position fell within a Michigan State University-annotated gene (http://rice.plantbiology.msu.edu/), the feature was determined (intron, exon, 3' or 5' UTR), and the name and function of the gene were retrieved. Call rates were measured for all markers, and markers with call rates below 80% were discarded. The allele frequency of the remaining markers was then calculated, and markers for which the minor allele had a frequency below 2.5% were also discarded.

### Imputation of missing genotypic data

Markers for which the minor allele frequency (MAF) is below 2.5% were discarded before imputing missing data. Missing data were estimated using Beagle v4.0 [44]. Beagle uses a localized haplotype cluster model. It is a special class of directed acyclic graph which empirically models haplotype frequencies on a local scale and therefore adapts to local structure in the data. The model determines a hidden Markov model that can be used to find the most likely haplotype pair for each individual, given the genotype data for that individual and the graphical haplotype frequency model. The method works iteratively using an expectation maximization type approach. The imputed missing data, probabilities of missing genotypes and inferred haplotypes are calculated from the model that is fitted at the final iteration.

### Analysis of population structure

Different statistical methods were employed to infer the number of subgroups in the panel. First, a model based Bayesian clustering analysis method implemented in STRUCTURE software version 2.3.4 [45] was used. The program was run with the following parameters: *k*, the number of groups in the panel varying from 1 to 9; 10 runs per *k* value; for each run, 10,000 burnin iterations followed by 10,000 MCMC (Markov Chain Monte Carlo) iterations. The optimal number of groups (*k*) was determined by Δ*k* following the method described in Evanno et al. [46]. STRUCTURE analysis was conducted for different numbers of markers evenly distributed on the whole genome. Very similar results were obtained when the number of markers was more than 1000. Second, a multi-dimensional scaling and cluster analysis method implemented in Awclust package [47] was used. Third, Tdiscriminant analysis of principal components (DAPC) method implemented in adegenet package [48] was used. The “find.cluster” function was used to identify clusters (*k*) and the optimal *k* value was determined according to the Bayesian Information Criterion (BIC), then the “dapc” function was used to check the classification quality.

### Kinship coefficient

Three methods were used to calculate kinship coefficient matrix (K). (1) Pairwise_IBS: the distance between two accessions is computed as the proportion of SNP which are different. The IBS is computed by subtracting all values of the distance matrix from 2 and then scaling the resultant matrix so that the minimum value is 0 and the maximum value is 2. It is implemented in TASSEL 5.2.6 [49]. (2) The scaled_IBS: the IBS was scaled to have the mean diagonal element equal to 1+F, where F is the inbreeding coefficient of the current population. It is the default method of the TASSEL 5.2.6 [49]. (3) The VanRaden method: the realized relatedness between individuals is computed according to ZZ'/(2∑*p*_*i*_(1-*p*_*i*_)) where Z = W-P, W is the marker matrix, P contains the allele frequencies multiplied by 2, *p*_*i*_ is the allele frequency of marker i, and the sum is over all loci [50]. It is the default method of GAPIT [51].

### Linkage disequilibrium

LD was measured by squared allele frequency correlations (r^2^) values between the pairs of markers using TASSEL 5.2.6 [49]. Only markers with MAF greater than 10% were used. Marker pairs were discretized into bins of 10 kb and the median r^2^ value was used as the estimate of r^2^ of a bin. The estimated r^2^ values were formulated as a function of physical distances between markers. A power law curve (y = ax^k^) was fitted to determine the physical position (x) corresponding to a given r^2^ value (y) [52]. The following scheme was used to estimate the background LD beyond which LD was assumed to be caused by genetic linkage: One marker per chromosome was randomly sampled and the maximum of all the pairwise r^2^ values was taken. The median of the maximum r^2^ values of 10,000 samples was taken as the basal r^2^ value. The physical distance at which r^2^ reached to the basal value was determined as the LD decay distance of the population. All analysis was done for the whole population and the three subpopulations inferred by STRUCTURE.

### Association analysis

All association models under the unified model for association mapping [53] can be described by considering how the two factors, the population structure (Q) and genetic relatedness between genotypes (K), are considered. We used four options for handling Q and four options for handling K to create 16 models. The four options for Q were: no Q, Q3 derived from STRUCTURE, Q7 derived from STRUCTURE and C7 derived from adegenet. The four options for K were: no K, K computed as pairwise_IBS (Kp scaled_IBS (Ks) and VanRaden method (Kv). All analyses were conducted using TASSEL 5.2.6 [49]. For models without K, known as general linear model (GLM) 1,000 permutations were used. For models with K, known as mixed liner model (MLM), the compressed mixed linear model approach [53, 54] and P3D [54] algorithms were adopted to reduce computing time. The best model for each trait-environment combination was chosen using the mean square difference (MSD) between observed and expected p-values of all marker loci, as suggested by Stich et al. [55]. MSD was a measure for the deviation of the observed p-values from the uniform distribution. Model with a smaller MSD is more appropriate. The critical p-value for declaring significant MTA was set to 0.0001.

## Results

### Basic statistics of markers

**Fig 1:**
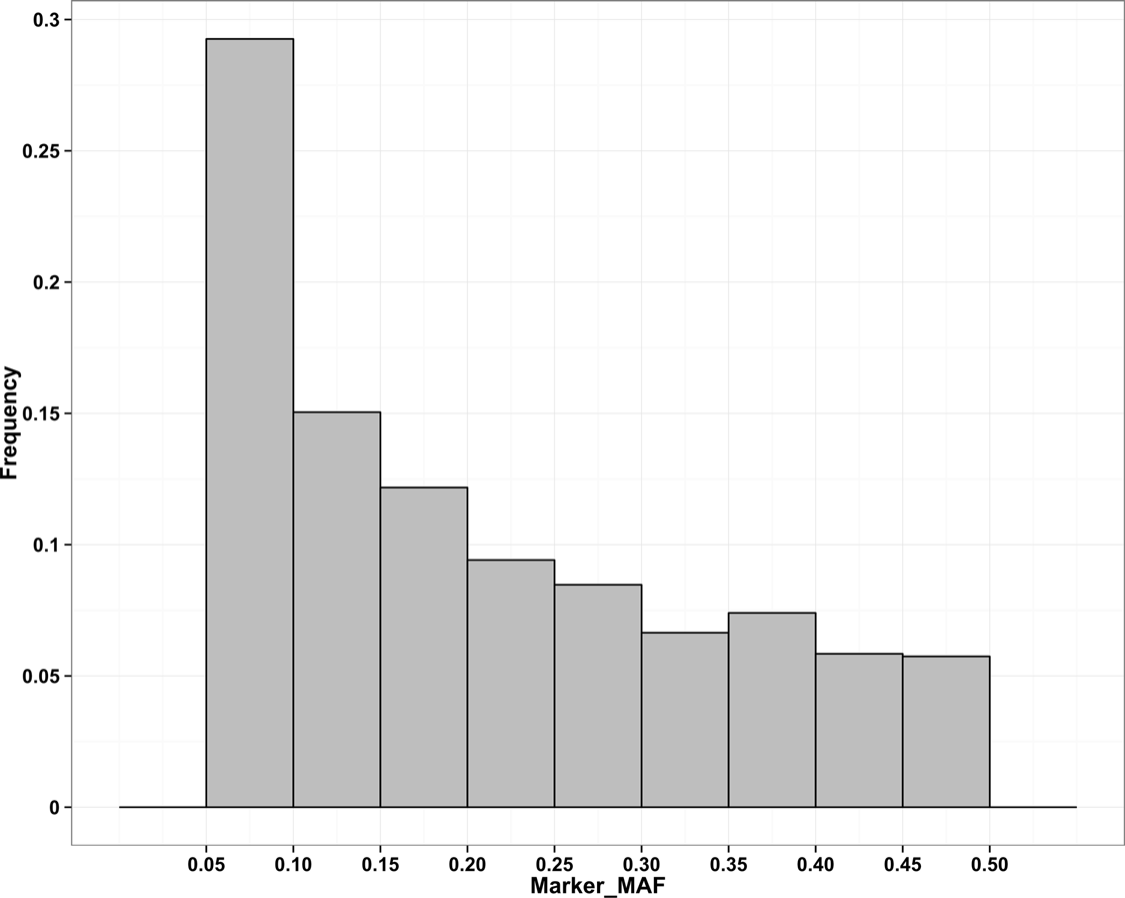
Frequency of markers in different MAF classes. SNP with MAF < 0.05 were excluded.

A total of 22,266 polymorphic markers were detected in the panel including 9,340 SNP and 12,926 DArT markers. By removing markers with MAF less than 5%, 18,824 high quality markers (7,885 SNP and 10,939 DArT) were used in association analysis. The number of markers per chromosome ranged from 891 on chromosome 10 to 2,361 on chromosome 1. The size of chromosome varied from 22.8Mb for chromosome 9 to 43.2 Mb for chromosome 1. The whole genome size was 371.7 Mb and the average distance between neighboring markers (marker spacing) was 20.2 kb. The average marker spacing ranged from 16.3 kb for chromosome 11 to 25.9 kb for chromosome 10 (S2 Table). About 70.4% of the neighboring markers distances were below the average value and 97.4% were less than 100 Kb. There were eight gaps devoid of markers larger than 500 kb on chromosomes 1 (D01_26116212—S01_26770440), 2 (D02_13852683—D02_15083642), 4 (D04_8765494—D04_9302397 and D04_16774867—D04_17319136), 6 (D06_18215124—D06_18803206), 7 (D07_11700243—S07_12785983 and S07_13836518—D07_14505070) and 11 (D11_11988263—S11_12639397). More than half of the markers (55.7%) had MAF lower than 0.20 (Fig 1).

### Phenotypic variation and trait correlation

**Fig 2:**
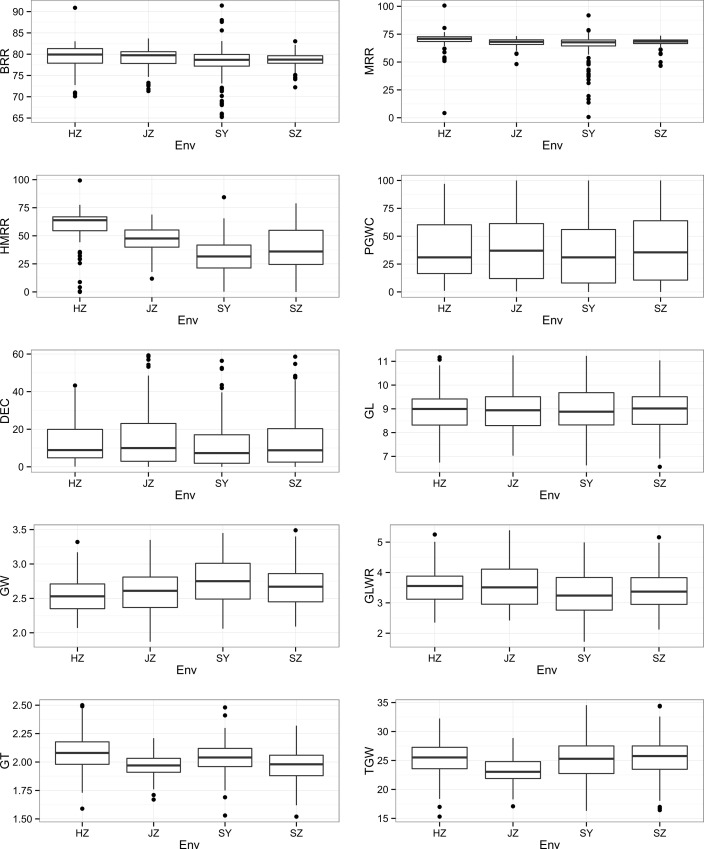
Box plot of 10 rice grain appearance and milling quality traits in four environments. HZ: Hangzhou. JZ: Jingzhou. SY: Sanya. SZ: Shenzhen. GL: Grain Length. GW: Grain Width. GLWR: Grain Length and Width Ratio. GT: Grain Thickness. TGW: Thousand Grain Weight. BRR: Brown Rice Rate. MRR: Milled Rice Rate. HMRR: Head Milled Rice Rate. PGWC: Percentage of Grains With Chalkiness. DEC: Degree of Endosperm chalkiness.

In general, the panel revealed a wide range variation for all the evaluated traits (Fig 2). Most of the traits appeared to be normally distributed, but some trait-environment combinations showed skewed distributions. For instance, the distributions of DEC in all environments, HMRR and PGWC in HZ and BRR in JZ were seriously skewed (Fig 2). Variance components derived from across-site analysis were given in Table 2. For appearance quality traits, the V_G_ was much larger than V_GEI_, indicating that GEI was negligible. For BRR, MRR and HMRR, the V_GEI_ was much larger than V_G_, suggesting that GEI was very important. However, the V_e_ for HMRR was very large, indicating phenotyping precision was low. The h^2^ for the appearance quality traits were high ranging from 0.82 for PGWC to 0.98 for GL and GLWR, while were low for the milling quality traits ranging from 0.19 for MRR to 0.27 for HMRR.

**Table 2:** Variance components and heritabilty estimated for 10 quality traits by across-site analysis.

Trait	V_E_	V_G_	V_GEI_	V_e_	Rep(Env)	h^2^
GL	0.00	0.62	0.00	0.08	0.01	0.98
GW	0.00	0.07	0.00	0.02	0.01	0.97
GLWR	0.00	0.32	0.00	0.05	0.01	0.98
GT	0.00	0.02	0.00	0.01	0.01	0.94
TGW	0.97	8.72	1.92	0.29	0.01	0.94
PGWC	0.62	86.04	59.81	27.7	1.72	0.82
DEC	0.00	465.84	277.44	138.17	6.72	0.84
BRR	0.08	0.57	5.57	0.97	0.31	0.27
MRR	3.01	2.75	44.98	4.71	0.48	0.19
HMRR	108.45	13.64	67.41	230.5	37.29	0.23

V_E_: environment variance; V_G_: genotype variance; V_GEI_: genotype- by- environment interaction variance; V_e_: residual variance; Rep(Env): replication variance within environment; h^2^: narrow-sense heritability.

**Fig 3:**
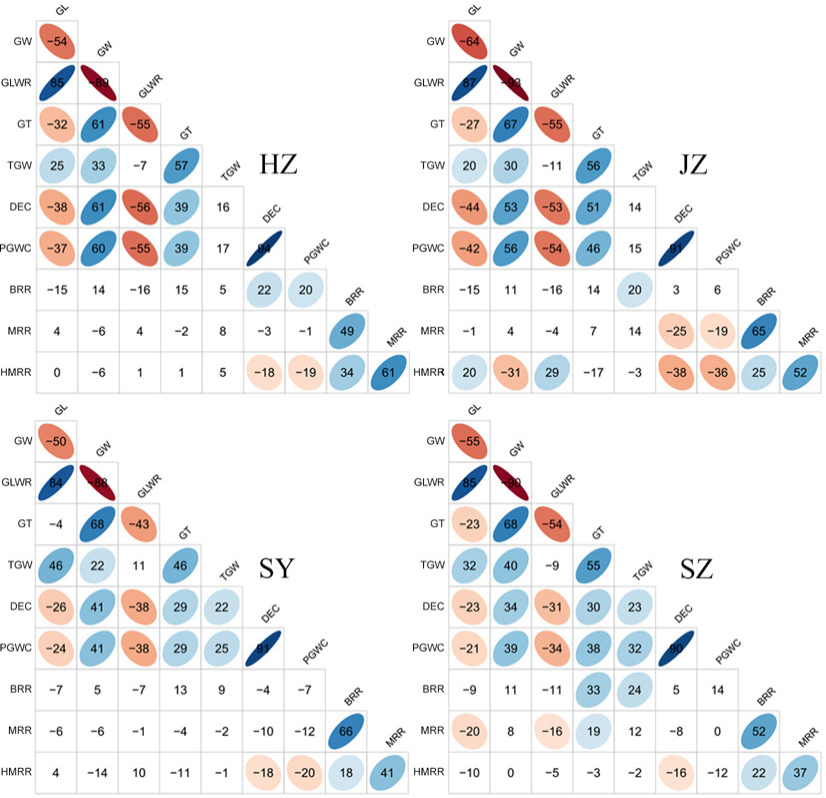
Correlations between 10 gain quality traits measured in HZ, JZ, SY and SZ. The values given were correlation coefficients multiplied by 100. The values without glyphs indicated insignificant at the level of 0.05.

The phenotypic correlations between traits were similar in the four testing environments. Low correlations were found between grain appearance quality traits and milling quality traits in all environments. GL showed a moderate and negative correlation with GW and a low and negative correlation with GT. Correlation between GW and GT was moderate and positive. TGW positively correlated with GL, GW and GT. High correlation was found between PGWC and DEC in all environments with coefficient ranging from 0.90 in SZ to 0.94 in HZ. The correlation between TGW and GT was moderate in all environments ranging from 0.46 in SY to 0.57 in HZ. Negative and moderate correlation was found between PGWC and HMRR. The correlation between PGWC and DED was strong and positive. The correlations between the three milling quality traits were positive ranging from low to moderate (Fig 3).

### Population structure

According to the value of Δ*k*, there were three groups in the current panel (S1 Fig). Group-I consisted of 88 accessions from 21 countries with 36 from China (36). Group-II had 96 accessions from 19 countries with 30 and 23 from Philippines and Senegal. Group-III had 88 accessions from 13 countries with more accessions from Madagascar (28), India (20) and Sri Lanka (19) (S2 Fig).

Hierarchical cluster analysis using Awclust suggested that the best number of clusters was also three based on Gap stastics (S1 Fig). The classification of accessions into groups was similar to that derived from STRUCTURE. Fifteen accessions of the group Q3-2 of STRUCTURE were assigned to the C3-3 of Awclust (S3 Fig).

The “find.clusters” function of adegenet showed a clear decrease of BIC until *k* = 7 clusters, after which BIC increased (S1 Fig). In this case, the elbow in the curve also matched the smallest BIC, and clearly indicated that seven clusters should be retained. Roughly speaking, the three groups detected by STRUCTURE were divided into two, two and three clusters by adegenet (S3 Fig). If we also chose seven groups for STRUCTURE analysis the Q3-1, Q3-2 and Q3-3 were further divided into two, two and four groups (S3 Fig). There were some differences in the assignments of accessions between *k* = 7 in STRUCTURE and adegent. The biggest difference was that the C7-4 of adegenet was distributed over five of the seven groups of STRUCTURE (S3 Fig).

### Whole genome patterns of LD decay

**Fig 4:**
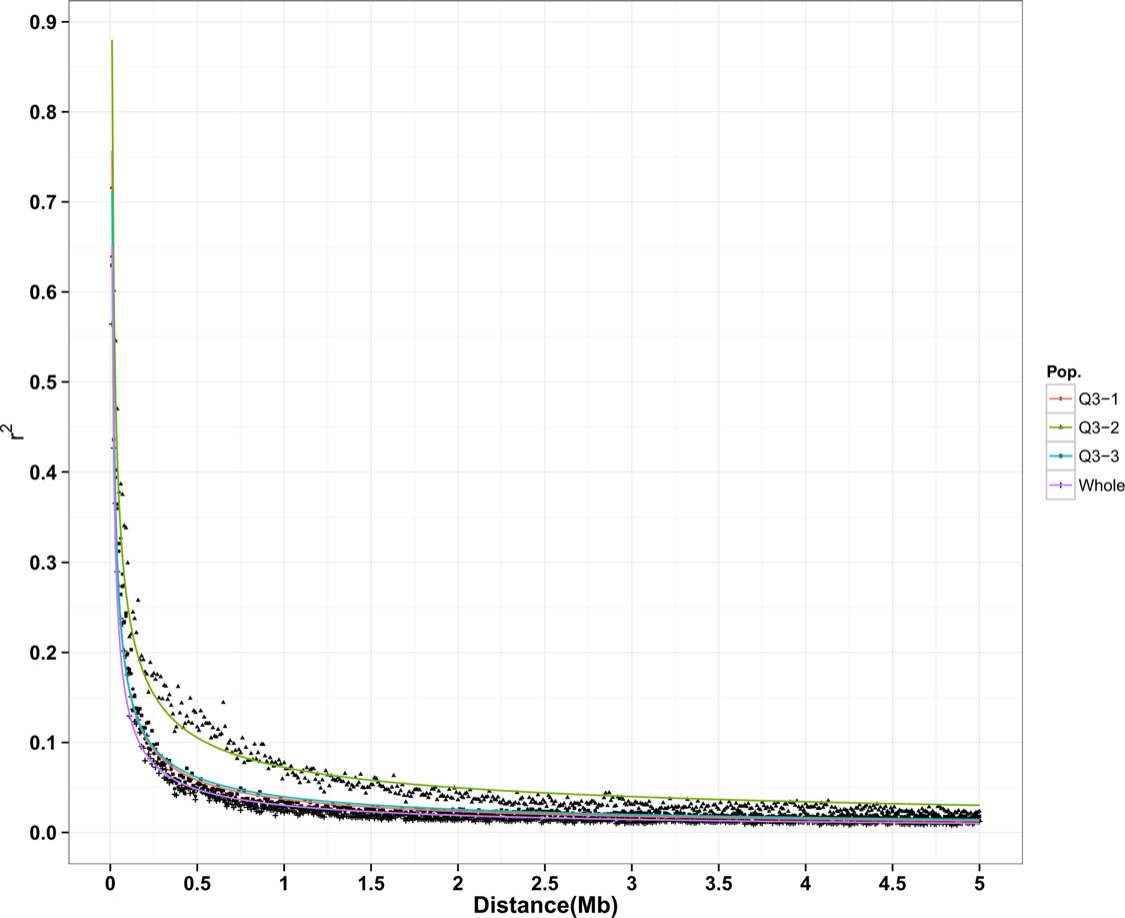
LD decay plot in the whole population and three subpopulations inferred by STRUCTURE. Y axis is the average r^2^ value of each 10 kb region and X axis is physical distance between markers in unit of Mb. A power law curve (y = ax^k^) was fitted to determine the physical position (x) corresponding to a given r^2^ value (y)

The decay of LD along physical distances was computed for both the whole population (272 accessions) and three subpopulations inferred by STRUCTURE *k* = 3. The determination coefficients (R^2^) of regression models were 0.87, 0.93, 0.88 and 0.95 for the whole population, Q3-1, Q3-2 and Q3-3, respectively (S3 Table). The maximum r^2^ was observed in the 0–10 kb marker interval for the whole population and subpopulations. The maximum r^2^ was lower in the whole population (0.65) than in the subpopulations. Q3-2 had the largest maximum r^2^ than Q3-1 (0.76) and Q3-3 (0.71). The basal r^2^ was 0.11, 0.16, 0.16 and 0.19 for the whole population, Q3-1, Q3-2 and Q3-3, respectively (S3 Table). The LD decay distance was 150 kb for the whole population, which was longer (slower) than those for Q3-1 (110 kb) and Q3-3 (80 kb) but shorter (faster) than that for Q3-2 (240 kb) (Fig 4 and S3 Table).

### Association analysis

#### Comparison of models

**Fig 5:**
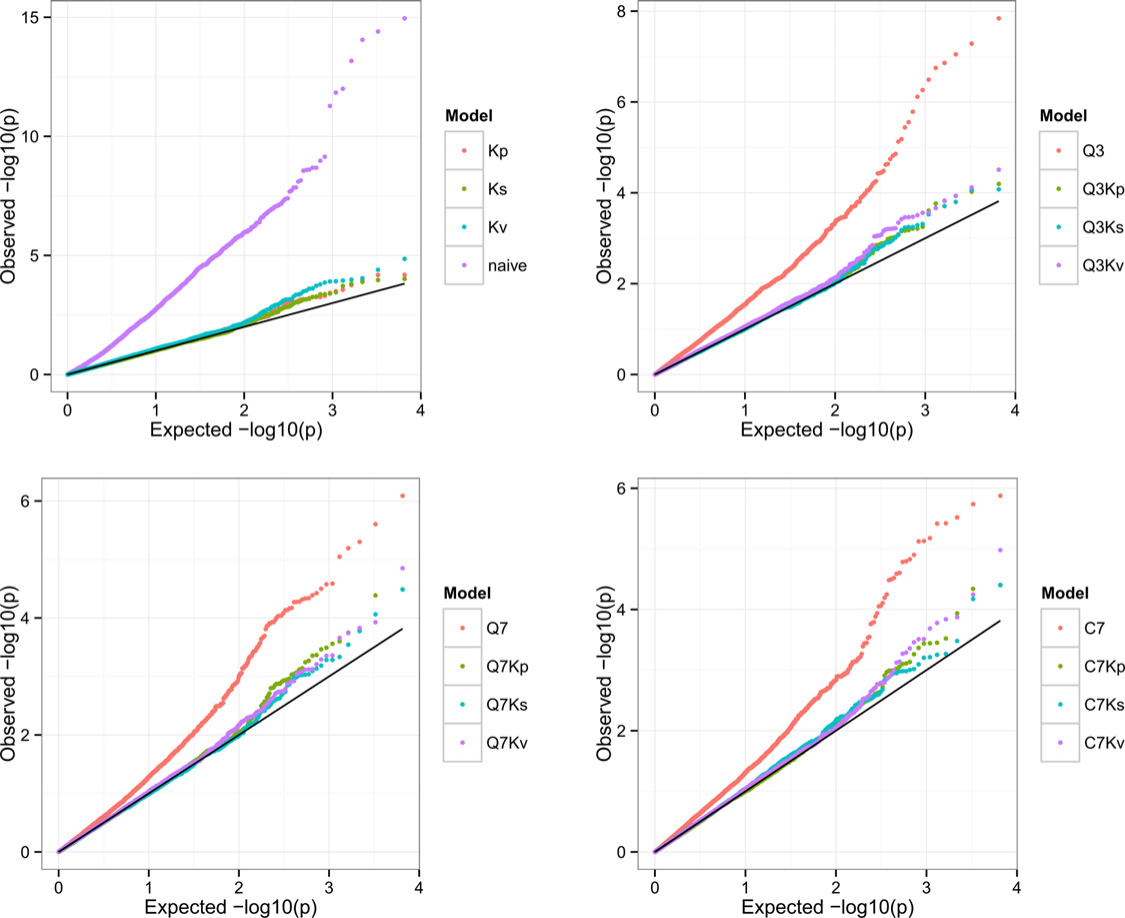
Quantile-quantile plots of 16 models for GL measured in HZ. The horizontal and vertical axes are -log10 transformed expected p-values and observed p-values, respectively. Model with more uniformly distributed p-values is better.

A total of 16 models for detecting associations were compared to choose the best model for each of the trait-environment combinations using MSD. The complete MSD results were given in S4 Table. Fig 5 gave the quantile-quantile plots for the 16 models for GL measured in HZ to illustrate the relative differences between models. The best model varied with traits and environments (S4 Table). For all the trait-environment combinations except BRR in SY, the naïve model was always inferior to other models (S4 Table), indicating complex population structure and genetic relatedness between accessions present in our panel affected the detection of MTA. According to the average MSD value across all trait-environment combinations, the Q models (Q3, Q7 and C7)that corrected for population structure only, were inferior to the K models (Kp, Ks and Kv) that corrected for the genetic relationships between accessions and the QK models (Q3Kp, Q3Ks, Q3Kv, Q7Kp, Q7Ks, Q7Kv, C7Kp, C7Ks and C7Kv) that corrected for both of population structure and genetic relationship (S4 Table). The QK model was the best model for most of the trait-environment combinations. The Q7 model was slightly better than the other two Q models. The Kp model was better than the Ks model and Kv model. The C7Kp model was better than the other QK models. For the models with the same Q but different K, the Q-Kp and Q-Ks models were similar and were slightly better than the Q-Kv model. The models with the same K but different Q had very similar MSD values. In the following sections only the MTA identified using the best model for each of the trait-environment combinations were presented and discussed.

#### Marker-trait associations

A total of 74 markers on all 12 chromosomes were found to be significantly associated with the 10 measured traits. By delineating significant markers with LD higher than 0.2 into a single QTL, the 74 markers were grouped into 38 QTL (Table 3).

**Table 3:** QTL identified for 10 traits in four environments.

QTL	Env	Marker	Alleles^a^	MAF	p	Effect^b^	R^2^ (%)^c^	Known gene/QTL
*qGL2*.*1*	SY	D02_25608957	T/A	0.16	5.93E-05	0.43	7.5	
*qGL2*.*2*	SY	S02_30573143	T/A	0.36	7.03E-05	0.5	7.4	
	SZ	S02_30573143	T/A	0.35	5.04E-05	0.43	7.0	
	HZ	S02_30664746	G/T	0.30	2.63E-05	0.51	8.1	
	SZ	S02_30664746	G/T	0.29	7.44E-05	0.44	6.7	
	HZ	S02_30664811	C/T	0.30	2.63E-05	0.51	8.1	
	SZ	S02_30664811	C/T	0.29	7.44E-05	0.44	6.7	
*qGL3*.*1*	HZ	S03_16663793	T/C	0.30	2.97E-05	0.79	8.0	*GS3*
	SY	S03_16663793	T/C	0.28	3.26E-06	0.98	10.2	
	SZ	S03_16663793	T/C	0.29	2.19E-05	0.69	7.7	
	SZ	S03_16731182	C/T	0.41	2.42E-05	-0.46	7.6	
	SZ	S03_16748937	C/T	0.47	3.92E-06	0.57	9.2	
	SZ	S03_16762099	A/G	0.47	1.13E-05	0.53	8.3	
	HZ	S03_16858510	C/G	0.45	7.31E-05	0.61	7.2	
	SY	S03_16858510	C/G	0.42	2.00E-05	0.69	8.5	
	SZ	S03_16858510	C/G	0.43	8.03E-07	0.65	10.5	
	SZ	S03_16996600	C/A	0.38	4.74E-05	0.48	7.0	
	SZ	S03_17000111	C/T	0.39	1.80E-05	0.5	7.9	
*qGL5*.*1*	SY	S05_5368086	C/T	0.38	8.44E-05	-0.48	7.2	*qSW5*
	SY	S05_5368151	A/G	0.38	8.10E-05	-0.48	7.2	
	SY	S05_5369527	A/T	0.37	5.71E-05	-0.49	7.5	
*qGL7*.*1*	HZ	S07_22019132	G/A	0.05	3.51E-05	-1.04	7.8	
	SY	S07_22019132	G/A	0.05	4.85E-05	-1.11	7.7	
	SZ	S07_22019132	G/A	0.05	9.95E-05	-0.81	6.4	
	SY	S07_22087092	T/A	0.13	6.54E-05	-0.66	7.4	
	HZ	S07_22099651	A/G	0.05	3.51E-05	-1.04	7.8	
	SY	S07_22099651	A/G	0.05	4.85E-05	-1.11	7.7	
	SZ	S07_22099651	A/G	0.05	9.95E-05	-0.81	6.4	
*qGL7*.*2*	SY	S07_22844850	C/T	0.18	6.30E-05	-0.63	7.5	*qGRL7*.*1*
*qGL9*.*1*	JZ	S09_10774239	A/T	0.50	7.39E-05	0.53	11.7	
*qGL11*.*1*	JZ	S11_2576141	T/G	0.35	5.76E-05	-0.57	12.0	*CycT1;3*
	SY	S11_2576141	T/G	0.35	6.41E-06	-0.63	9.6	
*qGW4*.*1*	SZ	S04_7167748	C/T	0.42	5.91E-05	0.16	6.8	
*qGW5*.*1*	SY	S05_5368086	C/T	0.38	1.94E-05	0.19	8.7	*qSW5*
	SZ	S05_5368086	C/T	0.35	3.26E-07	0.21	11.2	
	JZ	S05_5368151	A/G	0.39	1.06E-05	0.23	16.0	
	SY	S05_5368151	A/G	0.37	8.43E-06	0.19	9.5	
	SZ	S05_5368151	A/G	0.35	1.57E-07	0.22	11.9	
	JZ	S05_5369527	A/T	0.38	9.45E-06	0.24	16.2	
	SY	S05_5369527	A/T	0.37	5.09E-06	0.2	10.0	
	SZ	S05_5369527	A/T	0.34	5.54E-08	0.22	12.8	
*qGW7*.*1*	JZ	S07_19590107	G/C	0.08	4.41E-05	0.29	13.6	
	JZ	S07_19592078	T/A	0.08	4.41E-05	0.29	13.6	
*qGW10*.*1*	SZ	S10_14195109	A/T	0.05	7.54E-05	0.36	6.6	
	SZ	S10_14262980	A/G	0.05	7.54E-05	0.36	6.6	
*qGW11*.*1*	HZ	D11_7124485	T/A	0.17	8.76E-05	0.12	7.0	
*qGLWR1*.*1*	HZ	S01_5811755	A/T	0.08	4.83E-05	0.52	7.4	
	SZ	S01_5811755	A/T	0.08	9.32E-05	0.48	5.8	
*qGLWR3*.*1*	SZ	D03_14077712	T/A	0.15	9.18E-05	-0.4	5.8	
*qGLWR3*.*2*	SY	S03_16858510	C/G	0.42	9.44E-05	0.38	6.3	*GS3*
	SZ	S03_16858510	C/G	0.43	4.06E-05	0.36	6.4	
*qGLWR5*.*1*	JZ	S05_5368086	C/T	0.40	4.03E-05	-0.46	12.9	*qSW5*
	SY	S05_5368086	C/T	0.39	1.47E-06	-0.35	9.7	
	SZ	S05_5368086	C/T	0.35	3.09E-05	-0.31	6.6	
	JZ	S05_5368151	A/G	0.39	2.30E-05	-0.48	13.8	
	SY	S05_5368151	A/G	0.38	4.80E-07	-0.37	10.7	
	SZ	S05_5368151	A/G	0.35	1.42E-05	-0.33	7.2	
	HZ	S05_5369527	A/T	0.36	4.95E-05	-0.32	7.4	
	JZ	S05_5369527	A/T	0.38	1.96E-05	-0.48	14.1	
	SY	S05_5369527	A/T	0.38	1.97E-07	-0.38	11.4	
	SZ	S05_5369527	A/T	0.34	9.05E-06	-0.34	7.6	
*qGLWR7*.*1*	SY	S07_25383179	T/C	0.07	5.93E-05	0.5	6.6	*Srs1*
*qGLWR11*.*1*	JZ	S11_2576141	T/G	0.35	1.03E-05	-0.42	15.1	*CycT1;3*
*qGT1*.*1*	HZ	D01_2405901	A/T	0.10	2.77E-05	0.14	9.4	
*qGT4*.*1*	SZ	S04_29442031	T/C	0.16	6.57E-05	-0.13	7.0	
	SZ	S04_29495556	T/C	0.16	6.57E-05	-0.13	7.0	
*qGT5*.*1*	HZ	S05_5368086	C/T	0.33	2.37E-06	0.13	12.0	*qSW5*
	SZ	S05_5368086	C/T	0.35	1.13E-08	0.13	14.9	
	HZ	S05_5368151	A/G	0.33	9.02E-07	0.14	13.1	
	JZ	S05_5368151	A/G	0.39	8.98E-05	0.09	8.6	
	SZ	S05_5368151	A/G	0.35	4.47E-09	0.14	15.8	
	HZ	S05_5369527	A/T	0.32	8.77E-07	0.13	13.1	
	SY	S05_5369527	A/T	0.37	1.70E-05	0.09	8.5	
	SZ	S05_5369527	A/T	0.34	4.91E-09	0.14	15.7	
*qGT5*.*2*	SZ	S05_18307877	G/C	0.21	8.96E-05	-0.11	6.7	
	SZ	S05_18314309	C/T	0.21	8.96E-05	-0.11	6.7	
	SZ	S05_18321742	A/G	0.21	8.96E-05	-0.11	6.7	
*qTGW2*.*1*	SZ	S02_30688426	C/A	0.30	8.86E-05	1.78	7.7	
*qTGW5*.*1*	HZ	S05_4808850	T/A	0.48	6.59E-05	-2.06	10.1	
	HZ	S05_4856967	A/T	0.47	6.88E-05	-2.07	10.0	
	HZ	S05_4880400	A/G	0.47	6.88E-05	-2.07	10.0	
	HZ	S05_4889259	C/T	0.47	6.88E-05	-2.07	10.0	
*qTGW5*.*2*	SZ	S05_5368086	C/T	0.33	5.38E-05	2.07	8.2	*qSW5*
	SZ	S05_5369527	A/T	0.32	2.66E-05	2.14	8.9	
*qTGW7*.*1*	HZ	S07_21628162	C/T	0.08	5.77E-05	-3.72	10.3	
*qTGW7*.*2*	SZ	S07_22684516	C/A	0.05	9.17E-05	-4.26	7.7	*qGRL7*.*1*
*qPGWC5*.*1*	SZ	S05_5368086	C/T	0.32	5.16E-08	28.21	15.9	*qSW5*
	SZ	S05_5368151	A/G	0.32	2.97E-07	26.78	14.0	
	JZ	S05_5369527	A/T	0.38	6.52E-05	24.33	10.9	
	SZ	S05_5369527	A/T	0.31	1.72E-08	29.41	17.1	
*qPGWC6*.*1*	SZ	S06_6372444	A/C	0.09	5.76E-06	35.63	10.8	
*qDEC1*.*1*	SZ	S01_5811836	A/C	0.17	8.66E-05	11.18	7.3	
	SZ	S01_5972775	T/G	0.23	7.96E-05	10.37	7.4	
*qDEC5*.*1*	HZ	S05_4180511	T/C	0.10	7.25E-05	11.03	10.3	
*qDEC5*.*2*	SY	S05_5368086	C/T	0.37	7.27E-05	7.89	7.5	*qSW5*
	SY	S05_5368151	A/G	0.36	4.18E-05	8.2	8.0	
	SY	S05_5369527	A/T	0.36	2.05E-05	8.59	8.7	
	SZ	S05_5369527	A/T	0.29	3.23E-05	9.75	8.3	
*qDEC5*.*3*	SZ	S05_23647280	A/G	0.35	4.93E-05	-8.5	7.9	
*qDEC9*.*1*	HZ	S09_16262697	T/C	0.07	4.02E-05	-16.93	11.1	
*qBRR2*.*1*	SZ	D02_8060607	A/T	0.26	2.96E-05	-1.02	8.2	
	SZ	D02_8075892	T/A	0.27	2.24E-05	-1.03	8.5	
*qBRR2*.*2*	JZ	D02_25652984	A/T	0.06	1.96E-05	-4.75	15.6	
*qBRR3*.*1*	HZ	S03_22356459	T/C	0.07	8.52E-05	3.84	10.6	
*qBRR4*.*1*	HZ	S04_32002470	C/T	0.09	3.59E-06	3.65	14.5	
*qBRR7*.*1*	HZ	S07_29497149	T/A	0.21	8.03E-05	2.31	10.7	
*qMRR1*.*1*	HZ	S01_41957924	G/A	0.07	4.44E-05	10.06	6.6	
	HZ	S01_41957989	G/T	0.07	4.44E-05	10.06	6.6	
	HZ	S01_41970208	T/A	0.07	4.44E-05	10.06	6.6	
*qMRR2*.*1*	JZ	D02_26022390	A/T	0.07	1.61E-06	-8.71	19.9	
	JZ	D02_26022455	A/T	0.07	1.61E-06	-8.71	19.9	
	JZ	D02_26163208	T/A	0.05	3.29E-07	-7.59	22.8	
	JZ	D02_26265084	A/T	0.05	5.22E-06	-7.04	17.8	
	JZ	D02_26312559	A/T	0.06	4.14E-05	-7.86	14.1	
*qMRR3*.*1*	SZ	S03_4636350	A/G	0.21	9.69E-05	-1.91	7.3	
*qMRR7*.*1*	JZ	S07_5075055	A/G	0.06	9.29E-05	-5.45	12.8	
	JZ	S07_5081323	T/A	0.06	9.29E-05	-5.45	12.8	
*qMRR11*.*1*	SY	D11_6895700	T/A	0.06	4.05E-05	-12.33	7.8	
*qHMRR3*.*1*	SZ	D03_31372694	T/A	0.40	2.85E-05	12.34	9.0	
	SZ	D03_31380514	A/T	0.48	1.67E-05	12.53	9.5	
*qHMRR4*.*1*	HZ	S04_28018950	T/C	0.05	3.92E-06	-26.22	14.9	
*qHMRR5*.*1*	SZ	S05_12426396	T/C	0.18	8.01E-05	15.42	7.9	
*qHMRR6*.*1*	HZ	D06_2712642	A/T	0.12	9.37E-05	-14.26	11.0	
*qHMRR8*.*1*	SZ	S08_2638229	G/A	0.12	3.16E-05	17.8	8.9	
*qHMRR12*.*1*	SZ	S12_26692818	C/T	0.36	1.79E-05	12.81	9.5	
	SZ	S12_26695253	G/A	0.32	3.18E-05	12.65	8.9	

^a^Major/Minor allele.

^b^Effect: Allelic effect with respect to the minor allele.

^c^R^2^: Phenotypic variation explained.

For GL, eight QTL were identified on chromosomes 2, 3, 5, 7, 9 and 11. *qGL2*.*1* (D02_25608957) was detected in SY and explained 7.5% of the phenotypic variation (R^2^ = 7.5%). *qGL2*.*2* (S02_305731433, S02_30664746 and S02_30664811) was detected in HZ, SY and SZ with R^2^ being 8.1%, 7.4% and 7.0%, respectively. *qGL3*.*1* (S03_16663793, S03_16731182, S03_16748937, S03_16762099, S03_16858510, S03_16996600 and S03_17000111) was identified in HZ, SY and SZ with R^2^ being 8.0%, 10.2% and 10.5%, respectively. *qGL5*.*1* (S05_5368086, S05_5368151 and S05_5369527) was detected in SY with a R^2^ of 7.5%. The *qGL7*.*1* (S07_22019132, S07_22087092 and S07_22099651) was detected in HZ, SY and SZ and explained 7.8%, 7.7% and 6.4% of the phenotypic variance. *qGL7*.*2* (S07_22844850) was detected in SY with a R^2^ of 7.5%. *qGL9*.*1* (S09_10774239) was detected in JZ and explained 11.7% of the phenotypic variance. *qGL11*.*1* (S011_2576141) was detected in JZ and SY with R^2^ being 12.0% and 9.6%, respectively (Table 3).

For GW, five QTL on chromosomes 4, 5, 7, 10 and 11 were identified. *qGW4*.*1* (S04_7167748) was detected in SZ with a R^2^ of 6.8%. *qGW5*.*1* (S05_5368086, S05_5368151 and S05_5369527) was detected in JZ, SY and SZ with R^2^ being 16.2%, 10.0% and 12.8%, respectively. *qGW7*.*1* (S07_19590107 and S07_19592078) was detected in JZ with a R^2^ of 13.6%. *qGW10*.*1* (S010_14195109 and S010_14262980) was detected in SZ with a R^2^ of 6.6%. *qGW11*.*1* (D011_7124485) was detected in SZ with a R^2^ of 7.0% (Table 3).

For GLWR, six QTL were identified on chromosomes 1, 3, 5, 7 and 11. *qGLWR1*.*1* (S01_5811755) was detected in HZ and SZ with R^2^ being 7.4% and 5.8%, respectively. *qGLWR3*.*1* (D03_14077712) was detected in SZ with a R^2^ of 5.8%. *qGLWR3*.*2* (S03_16858510) was detected in SY and SZ with R^2^ being 6.3% and 6.4%, respectively. *qGLWR5*.*1* (S05_5368086, S05_5368151 and S05_5369527) was detected in HZ, JZ, SY and SZ with R^2^ being 7.4%, 14.1%, 11.4% and 7.6%, respectively. *qGLWR7*.*1* (S07_25383179) was detected in SY with a R^2^ of 6.6%. *qGLWR11*.*1* (S011_2576141) was detected in JZ and explained 6.6% of the phenotypic variance (Table 3).

For GT, four QTL were found on chromosome 1, 4 and 5. *qGT1*.*1* (D01_2405901) was detected in HZ with a R^2^ of 9.4%. *qGT4*.*1* (S04_29442031 and S04_29495556) was detected in SZ with a R^2^ of 7.0%. *qGT5*.*1* (S05_5368086, S05_5368151 and S05_5369527) was detected in HZ, JZ, SY and SZ with R^2^ being 13.1%, 8.6%, 8.5% and 15.8%, respectively. *qGT5*.*2* (S05_18307877, S05_18314309 and S05_18321742) was detected in SZ with a R^2^ of 6.7% (Table 3).

For TGW, five QTL were found on chromosome 2, 5 and 7. *qTGW2*.*1* (S02_30688426) was detected in SZ with a R^2^ of 7.7%. *qTGW5*.*1* (S05_4808850, S05_4856967, S05_4880400 and S05_4889259) was detected in HZ with a R^2^ of 10.1%. *qTGW5*.*2* (S05_5368086 and S05_5369527) was detected in SZ with a R^2^ of 8.9%. *qTGW7*.*1* (S07_21628162) was detected in HZ with a R^2^ of 10.3%. *qTGW7*.*2* (S07_22684516) was detected in SZ with a R^2^ of 7.7% (Table 3).

For PGWC, two QTL on chromosomes 5 and 6 were identified. *qPGWC5*.*1* (S05_5368086, S05_5368151 and S05_5369527) was detected in JZ and SZ with R^2^ being 10.9% and 17.1%, respectively. *qPGWC6*.*1* (S06_6372444) was detected in SZ with a R^2^ of 10.8% (Table 3).

For DEC, five QTL were identified on chromosomes 1, 5 and 9. *qDEC1*.*1* (S01_5811836 and S01_5972775) was detected in SZ with a R^2^ of 7.4%. *qDEC5*.*1* (S05_4180511) was detected in HZ with a R^2^ of 10.3%. *qDEC5*.*2* (S05_5368086, S05_5368151 and S05_5369527) was detected in SY and SZ with R^2^ being 8.7% and 8.3%, respectively. *qDEC5*.*3* (S05_23647280) was detected in SZ with a R^2^ of 7.9%. *qDEC9*.*1* (S09_16262697) was detected in HZ with a R^2^ of 11.1% (Table 3).

For BRR, five QTL located on chromosomes 2, 3, 4 and 7 were detected. *qBRR2*.*1* (D02_8060607 and D02_8075892) was identified in SZ with a R^2^ of 8.5%. *qBRR2*.*2* (D02_25652984) was identified in SZ with a R^2^ of 15.6%. *qBRR3*.*1* (S03_22356459) was identified in HZ with a R^2^ of 10.6%. *qBRR4*.*1* (S04_32002470) was identified in HZ with a R^2^ of 14.5% (Table 3).

For MRR, five QTL located on chromosomes 1, 2, 3, 7 and 11 were detected. *qMRR1*.*1* (S01_41957924, S01_41957989 and S01_41970208) was identified in HZ with a R^2^ of 6.6%. *qMRR2*.*1* (D02_26022390, D02_26022455, D02_26163208, D02_26265084 and D02_26312559) was identified in JZ with a R^2^ of 22.8%. *qMRR3*.*1* (S03_4636350) was identified in SZ with a R^2^ of 7.3%. *qMRR7*.*1* (S07_5075055 and S07_5081323) was identified in JZ with a R^2^ of12.8%. *qMRR11*.*1* (D11_6895700) was identified in SY with a R^2^ of 7.8% (Table 3).

Six QTL were detected for HMRR. They were on chromosomes 3, 4, 5, 6, 8 and 12. *qHMRR3*.*1* (D03_31372694 and D03_31380514) was identified in SZ with a R^2^ of 9.5%. *qHMRR4*.*1* (S04_28018950) was identified in HZ with a R^2^ of 14.9%. *qHMRR5*.*1* (S05_12426396) was identified in SZ with a R^2^ of 7.9%. *qHMRR6*.*1* (D06_2712642) was identified in HZ with a R^2^ of 11.0%. *qHMRR8*.*1* (S08_2638229) was identified in SZ with a R^2^ of 8.9%. *qHMRR12*.*1* (S012_26692818 and S012_26695253) was identified in SZ with a R^2^ of 9.5% (Table 3).

Some of the QTL for different traits were in the same chromosomal regions and were regarded as the same QTL when the number of QTL was counted across traits. They were *qDEC1*.*1* and *qGLWR1*.*1* on chromosome 1; *qBRR2*.*2* and *qGL2*.*1* on chromosome 2; *qGL2*.*2* and *qTGW2*.*1* on chromosome 2; *qGLWR3*.*2* and *qGL3*.*1* on chromosome 3; *qGL5*.*1*, *qGW5*.*1*, *qGLWR5*.*1*, *qGT5*.*1*, *qTGW5*.*2*, *qPGWC5*.*1* and *qDEC5*.*2* on chromosome 5; *qGL7*.*2* and *qTGW7*.*2* on chromosome 7; *qGW11*.*1* and *qMRR11*.*1* on chromosome 11; and *qGLWR11*.*1* and *qGL11*.*1* on chromosome 11.

## Discussion

### Phenotypic variation and trait correlation

Great variation was observed for all traits in all testing environments in the present study, suggesting that the association panel can be effectively used for GWAS of various quality traits. Grain appearance quality traits, particularly grain size and weight traits had high heritability and showed only small GEI (Table 2), suggesting that breeding for a wide range of growing environments could be feasible and effective. Grain chalkiness and milling quality traits had low heritability and large contribution of GEI and as a result will be more difficult to improve through conventional selection breeding.

The relationship between grain quality traits in rice is complex. In the present study, positive correlations were observed between TGW and GL, GW and GT, which was consistent with previous studies [2, 26, 56]. The coefficients were moderate, which might be the result of negative correlations between GL and GW and GT. The correlation between GL and GW were -0.54, -0.64, -0.50 and -0.55 in HZ, JZ, SY and SZ, respectively. However, a weak and positive (0.11 in SY) or negative (-0.11, -0.07 and -0.09 in JZ, HZ and SZ, respectively) correlation was observed between TGW and GLWR, which was different with the result of Xu et al. [57]. They found TGW and GLWR had a moderate and positive correlation (0.44). Consistent with previous reports, BRR was negatively correlated with GL but positively correlated with GW, even though the correlation coefficients were small. In the process of milling, the long and slender rice grain is easier to break than short and round one. Chalkiness is also an important factor influencing rice milling traits especially HMRR. Chalky grains are also more prone to breakage at chalky area. We found HMRR was negatively correlated with PGWC and DEC, which was consistent with the study of Zheng et al. [58].

### LD pattern

The basal r^2^ was 0.11 and estimated LD decay distance was 150 kb for the whole population (S3 Table). The LD decay distance ranged from 80 kb to 240 kb in three subpopulations. This was in agreement with previous finding that cultivated rice has a long range LD from close to 100 kb to over 200 kb [59, 60]. Compared with the whole population, LD dropped much faster in Q3-1 and Q3-3 but more slowly in Q3-2. The effect of population structure on LD pattern has been widely reported [52, 60–62]. Varying patterns of LD decay in different subpopulations were likely reflecting their breeding histories and the origins of accessions in subpopulations [63] and may influence the QTL mapping resolution of the panel. Given that our average marker density (one marker per 20.2 kb) was higher than the r^2^ decay, we expected to have reasonable resolution and power to identify common variants of large effect associated with traits of interest.

### Model comparison

It is well-known that the results of association analysis using different models would be very different. However, there is no generally acceptable best model. It is likely that the best model differs among populations, traits and growing environments. The most appropriate model should be chosen by comparing different models. In the present study, MSD proposed by Stich et al. [55] was used to determine the best model for each of the 40 trait-environment combinations. The results showed that the naïve model and the Q models were almost always inferior to the K and QK models (S4 Table). The average MSD value across all trait-environment combinations of the naïve model was much higher compared to those of the Q models, indicating introduction of population structure could tremendously reduce false positives. Q derived from different numbers of subpopulations (*k*) also leaded to different results. According to MSD, increasing *k* from three to seven resulted in better control of false positives (S4 Table). When population structure was detected by different methods, the resultant Q and as a result the association results were different. For instance, the Q7 model, which used the Bayesian clustering analysis method implemented in STRUCTURE, was better than the C7 model, which used the Q from the DAPC method (S4 Table). It was clear from our results that the three Q models were rarely the best model and the K and QK models were better (S4 Table). This was consistent with most of the previous studies that showed that GLM method (Q model) was inferior to MLM method (K and QK models). A recent example in rice was the study ofCourtois et al. [41]. They performed association study for 16 root depth and associated traits in a panel of japonica accessions and chose the best model by comparing the likelihoods of each model using the BIC [64]. Their results showed that the QK model was the best model for 13 measured traits, the K model was the best for the other 3 traits, and the Q model was always inferior to the other two models.

For the models with the same Q but different K, the Q-Kp and Q-Ks models were similar and were slightly better than the Q-Kv model (S4 Table). The models with the same K but different Q had very similar MSD values, indicating that once the genetic relatedness between genotypes was well accounted, the differences between methods for inferring Q became less important. Therefore, when both Q and K were considered simultaneously, K played a more important role than Q and different K should be tried to determine the best model.

The best model differed for the same trait measured in different environments and different traits measured in the same environment (S4 Table). For traits with low heritability, the best model changed greatly across environments. For instance, BRR (h^2^ = 0.27), MRR (h^2^ = 0.19) and HMRR (h^2^ = 0.23), there was no common best model in the four environments. However, for traits with high heritability the same best model tended to be found across environments. For instance, Q7Kv was the best model for GL (h^2^ = 0.98) in HZ, JZ and SY and C7Kv was the best model for GW (h^2^ = 0.97) in HZ and SZ and Kv was the best model for GW in JZ and SY.

In the present study, we selected the model with the smallest MSD value as the best model. It was observed that models with very similar MSD values had very similar association results, indicating that MSD is a simple but effective method for choosing the appropriate model(s) in GWAS.

### The effects of environment and QEI

In the present study, the total number of identified QTL varied greatly across environments, indicating that testing environment had important influence on GWAS. Out of the 38 identified QTL only one (*qGLWR5*.*1* (*qGT5*.*1*)), four (*qGL2*.*2*, *qGL3*.*1*, *qGL7*.*1* and qGW5.1) and four QTL (*qGLWR1*.*1*, *qGL11*.*1*, *qPGWC5*.*1* (*qDEC5*.*2*) and qGLWR3.2) were detected in four, three and two environments, respectively (Table 3). Furthermore, even for *qGLWR5*.*1*, which was detected in all the four environments, the R^2^ varied from 7.4% to 14.1% across environments. These results may be partially attributed to QEI. For BRR, MRR and HMRR that showed significant GEI, all QTL were detected in only one of the four environments (Table 3). However, the big differences in population size across environments may also be partially responsible for the observed differences between environments. For most of the traits the population size was much larger in SY and SZ than in HZ and JZ (Table 1).

### GWAS is effective

In the present study, five out of the 38 identified QTL were adjacent to previously known genes or fine mapped QTL, suggesting that association mapping was an effective way to map QTL for rice grain quality traits. On chromosome 5, *qGL5*.*1*, *qGW5*.*1*, *qGLWR5*.*1*, *qGT5*.*1*, *qTGW5*.*2*, *qPGWC5*.*1* and *qDEC5*.*2* were located in genomic region (S05_5368086, S05_5368151 and S05_5369527) close to the cloned gene *qSW5* affecting GW and TGW [18]. *qSW5* was in 5362625bp - 5370506bp in the Os-Nipponbare-Reference- IRGSP-1.0 according to the complete cds sequence of *qSW5* in *indica*. The *qSW5* functioned as negative regulator of grain width and weight and a deletion in *qSW5* resulted in a significant increase in sink size owing to an increase in cell number in the outer glume of the rice flower and finally increased the grain width and weight [18]. The *qSW5* was also reported to regulate GL, GLWR and GT [65]. However, the effects of *qSW5* on PGWC and DEC have not been reported before. Our results suggested that *qSW5* could be used to increase GL and reduce DEC and PGWC simultaneously. The *qGL3*.*1* (S03_16663793, S03_16731182, S03_16748937, S03_16762099, S03_16858510, S03_16996600 and S03_17000111) and *qGLWR3*.*2* (S03_16858510) associated with GL and GLWR, were close to *GS3* (16729228–16735641) affecting grain shape. *GS3* functions as a negative regulator for grain size which is a major QTL for GL and TGW and a minor QTL for GW and GT [16]. The *qGL7*.*2* (S07_22844850) and *qTGW7*.*2* (S07_22684516) were located in the region harboring the previously fine mapped QTL affecting GL, GW and GLWR, *qGRL7*.*1* (22127.4Kb—24526.7Kb) [6]. The *qGLWR7*.*1* (S07_25383179) was located in the region of *Srs1* (25381698–25389547) affecting grain size [23]. The *srs1* results in small and round seed due to the reduction in both cell length and cell number in the longitudinal direction, and the elongation of the cells in the lateral direction of the lemma. The *qGL11*.*1* (S11_2576141) and *qGLWR11*.*1* (S11_2576141) were close to *CycT1;3* (2730143–2736649) whose down regulation results in a shorter grain.

Thirty-three of the identified QTL were located on chromosomal regions where no QTL for the measured traits have been reported and can be regarded as novel QTL. Five of the QTL including *qDEC1*.*1* (*qGLWR1*.*1*), *qBRR2*.*2* (*qGL2*.*1*), *qTGW2*.*1* (*qGL2*.*2*), *qGW11*.*1* (*qMRR11*.*1*) and *qGL7*.*1* affected multiple traits with relatively large effects and/or were detected in multiple environments.

### The number of QTL identified is small

Compared to the previous GWAS studies in rice the number of QTLs detected was small in the present study. Many of the known genes affecting traits measured were not detected in the present study. The most likely reason is the small population size. Most of the association panels had more than 400 accessions while the present panel only had 230 or fewer accessions. The effect of population size on GWAS is well known. It is particularly important for detecting loci with low MAF to use a large panel. Because the panel size was small markers with MAF <0.05 were filleted out before association analysis. The trait-affecting genes were missed out if markers in strong LD with them had low MAF in our panel. For example, the gene *PGL2* (LOC_Os02g51320) was involved in controlling grain length and weight of rice through interaction with a typical bHLH protein APG. Marker S02_31422564 very close to this gene (1.4 kb) was removed before association analysis since its MAF was only 0.02. The big differences between environments in population size could also explain why most of the QTL (53%) were detected in SZ. Nevertheless, loci with large effects were also detected for some of the traits with small population size. For instance, the population size of GW in JZ is only 132, but *qGW5*.*1* was identified with a high R^2^ of 16.2%. Therefore, provided that the MAF is high enough, major loci would still be detected even if the population size was small. Another possible reason is that the present panel consists of only *indica* accessions while most of the previous studies used panels with a few subspecicies/ecotypes. It is expected that genes that determine the major trait differences between subspecies/ecotypes could not be detected in our study but might be detectable in other studies. We also expected that more false positives were present when a panel with very diverse accessions was used. The use of a single subspecies allows controlling population structure at finer scale and as results reduces false positives. The critical significant threshold value used to declare significant association also influenced the analysis results. For instance, the *qGW5*.*1* was found to be significant in JZ, SY and SZ but not in HZ. By inspecting the p values in HZ, it could be seen that the p values of S05_5368086, S05_5368151 and S05_5369527, were 0.00065, 0.00035 and 0.00014, respectively, which were only slightly higher than the applied critical significant threshold 10^−4^. It might be worth pointing out that the number of markers used in the present study was smaller than or similar to the reported GWAS in rice. However, the marker density was sufficient for most of the genomic regions based on LD decay pattern. Furthermore, the eight gaps devoid of markers below critical LD did not harbor any known genes affecting the studied traits. Therefore, the relatively low marker density was unlikely a major contributor to the small number of detected QTL. Nevertheless, the marker density might be too low in some chromosomal regions where LD decays more rapidly or breaks down due to recombination events [41]. For instance, the *GW2* (LOC_Os02g14720) controlling grain width and weight, was in the marker interval of S02_8069581—S01_8181973. However, we could not identify any markers associated with GW or TGW in this region, since LD was very low (about 0.02). Additional markers in such regions are clearly needed.

## Conclusions

Wide ranges of genetic variations were present in the *indica* association panel for all 10 measured grain quality traits. The accessions could serve as sources of novel genes and alleles for improving rice appearance quality and milling quality traits. GWAS conducted using proper statistical models is a powerful method for elucidating the genetic basis of rice quality traits. Although it is generally true that MLM is better than the naïve model and GLM (Q) model for GWAS, the most appropriate model varies across traits and environments and must be chosen by comparing different models. MSD was found to be a simple but effective method for choosing the appropriate model in GWAS. Based on the selected best model for each of the trait-environment combinations, 38 QTL were identified for 10 rice grain appearance quality and milling quality traits including GL, GW, GLWR, GT, TGW, PGWC, DEC, BRR, MRR and HMRR. Eight genomic regions harbored QTL for more than one trait. Five QTL were found to concur with previously cloned genes or fine mapped QTL. Thirty-three were novel. Five of the novel QTL including *qDEC1*.*1* (*qGLWR1*.*1*), *qBRR2*.*2* (*qGL2*.*1*), *qTGW2*.*1* (*qGL2*.*2*), *qGW11*.*1* (*qMRR11*.*1*) and *qGL7*.*1* affected multiple traits with relatively large effects and/or were detected in multiple environments. Our results provided an insight into the genetic architecture of rice grain appearance quality and milling quality traits. The novel QTL are important candidates for future functional characterization studies.

## Supporting Information

S1 Fig Population structure analysis of 272 indica accessions. (a) Log probability change (Δk) as function of number of subpopulations (STRUCTURE). (b) Gap statistics as function of number of clusters (Awclust). (c) Value of BIC versus number of clusters (adegenet).
(TIF)

S2 Fig Population sub-structuring of 272 *indica* accessions based on 1083 SNP. (TIF)

S3 Fig Comparison of accession grouping of different statistical methods. (a) STRUCTURE k = 3 and Awclust k = 3. (b) STRUCTURE k = 3 and adegenet k = 7. (c) STRUCTURE k = 3 and k = 7. (d) STRUCTURE k = 7 and adegenet k = 7.
(TIF)

S1 Table Origin of 272 indica accessions of the association panel. (DOCX)

S2 Table Distributions of markers on chromosomes. (DOCX)

S3 Table Basic characteristics of LD pattern in the whole panel and three subpopulations. (DOCX)

S4 Table Comparison of 16 association models based the mean squared difference (MSD) between the observed and expected p values for 40 traits (10)-environment (4) combinations. (DOCX)
