# Supplementary figures and images for: Genome-Wide Association Study of Grain Appearance and Milling Quality in a Worldwide Collection of Indica Rice Germplasm
Source: PLoS One. 2015 Dec 29;10(12):e0145577. doi: 10.1371/journal.pone.0145577 (PMC4694703; doi:10.1371/journal.pone.0145577)

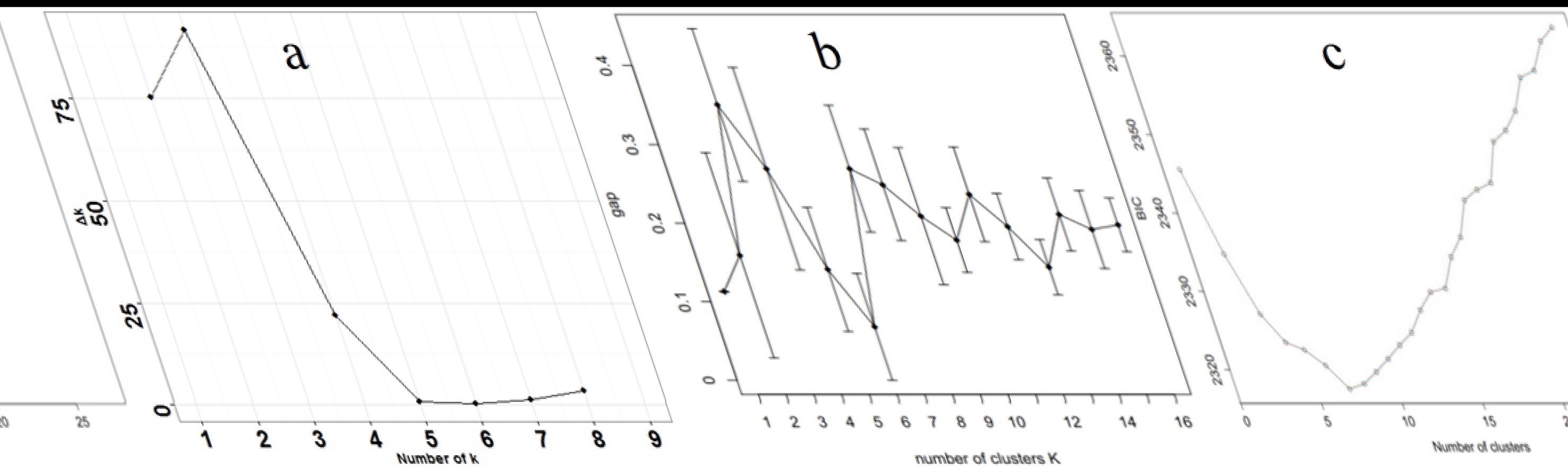

Supplement: S1 Fig — (a) Log probability change (Δk) as function of number of subpopulations (STRUCTURE). (b) Gap statistics as function of number of clusters (Awclust). (c) Value of BIC versus number of clusters (adegenet). (TIF) [file pone.0145577.s001.tif]

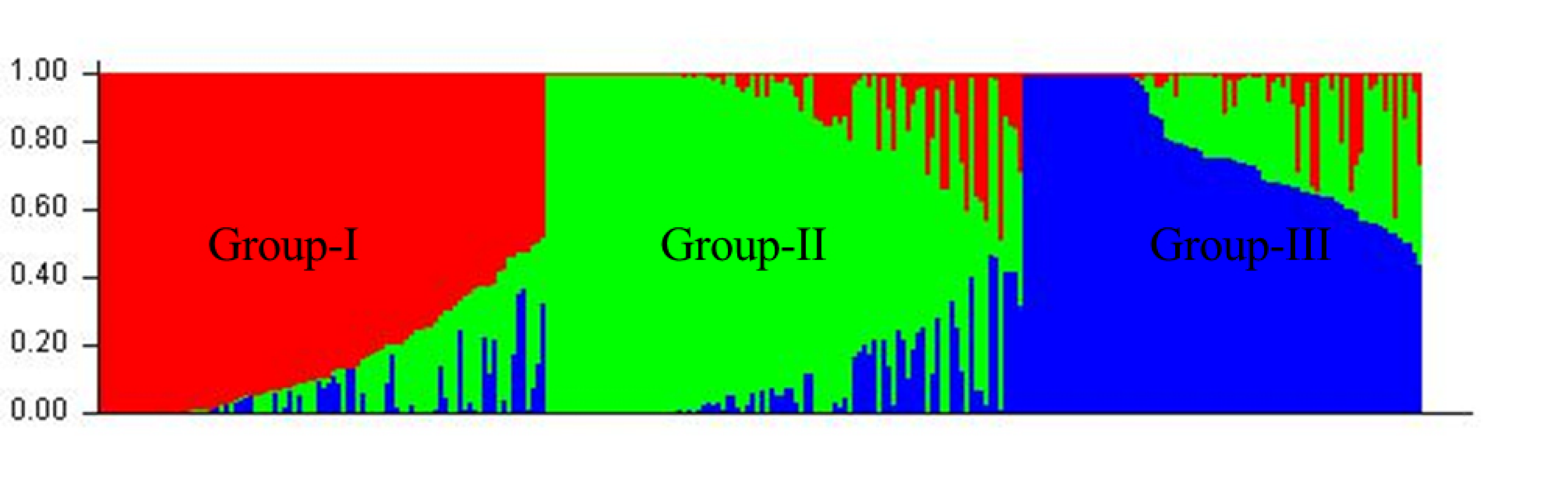

Supplement: S2 Fig — (TIF) [file pone.0145577.s002.tif]

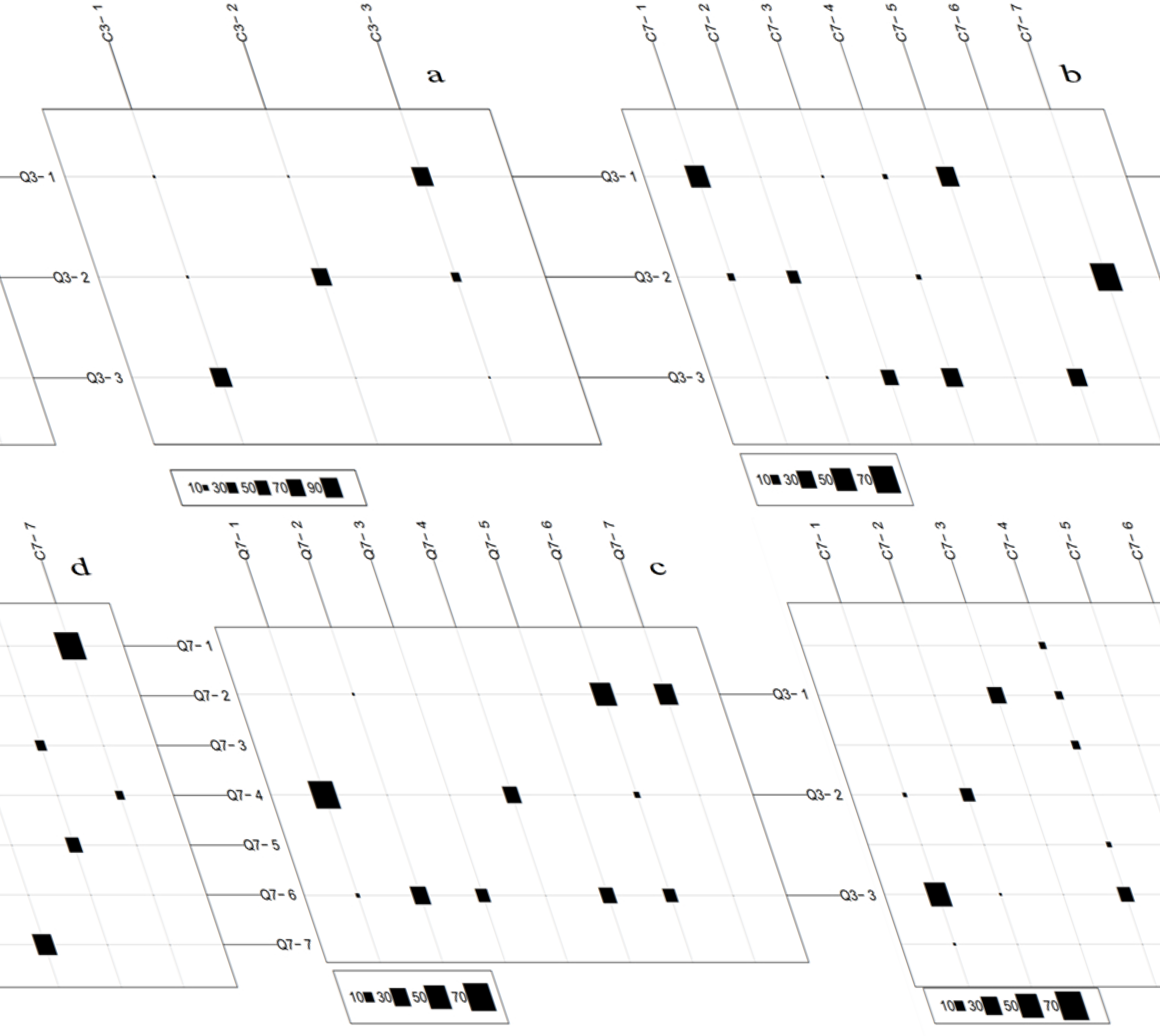

Supplement: S3 Fig — (a) STRUCTURE k = 3 and Awclust k = 3. (b) STRUCTURE k = 3 and adegenet k = 7. (c) STRUCTURE k = 3 and k = 7. (d) STRUCTURE k = 7 and adegenet k = 7. (TIF) [file pone.0145577.s003.tif]
