# Supplementary material for: Genome-Wide Association Study of Grain Appearance and Milling Quality in a Worldwide Collection of Indica Rice Germplasm
Source: PLoS One. 2015 Dec 29;10(12):e0145577. doi: 10.1371/journal.pone.0145577 (PMC4694703; doi:10.1371/journal.pone.0145577)
Supplement: S1 Table — (DOCX) [file pone.0145577.s004.docx]

**S1 Table. Origin of 272 *indica* accessions of the association panel.**

| IRGC No. | Origin | IRGC No. | Origin | IRGC No. | Origin | IRGC No. | Origin |
| --- | --- | --- | --- | --- | --- | --- | --- |
| 121159 | China | 120988 | Philippines | 121857 | Senegal | 122239 | Mali |
| 117793 | China | 122112 | Philippines | 122297 | Senegal | 121771 | Mali |
| 121137 | China | 122093 | Philippines | 121849 | Senegal | 121799 | Mali |
| 120970 | China | 122156 | Philippines | 121853 | Senegal | 121827 | Mali |
| 117280 | China | 121752 | Philippines | 122289 | Senegal | 121821 | Mali |
| 121156 | China | 121705 | Philippines | 122290 | Senegal | 122140 | Mali |
| 120862 | China | 120986 | Philippines | 121847 | Senegal | 121738 | Mali |
| 120909 | China | 121136 | Madagascar | 121852 | Senegal | 121642 | Viet Nam |
| 120880 | China | 121151 | Madagascar | 121848 | Senegal | 121043 | Viet Nam |
| 117636 | China | 117527 | Madagascar | 121858 | Senegal | 117574 | Viet Nam |
| 117681 | China | 121050 | Madagascar | 121850 | Senegal | 117622 | Viet Nam |
| 121157 | China | 121840 | Madagascar | 121851 | Senegal | 121104 | Viet Nam |
| 121063 | China | 121074 | Madagascar | 121855 | Senegal | 117682 | Viet Nam |
| 121040 | China | 121049 | Madagascar | 122292 | Senegal | 117478 | Viet Nam |
| 121092 | China | 121124 | Madagascar | 122291 | Senegal | 121100 | Bhutan |
| 120929 | China | 121836 | Madagascar | 122285 | Senegal | 121576 | Bhutan |
| 120914 | China | 120859 | Madagascar | 122284 | Senegal | 121099 | Bhutan |
| 121093 | China | 121073 | Madagascar | 121846 | Senegal | 121161 | Bhutan |
| 121110 | China | 121785 | Madagascar | 122288 | Senegal | 121015 | Bhutan |
| 120925 | China | 121823 | Madagascar | 122232 | Senegal | 121701 | Bhutan |
| 121164 | China | 122159 | Madagascar | 121024 | Sri Lanka | 117841 | Taiwan |
| 121176 | China | 121144 | Madagascar | 120892 | Sri Lanka | 117784 | Taiwan |
| 121028 | China | 117470 | Madagascar | 121079 | Sri Lanka | 117826 | Taiwan |
| 117745 | China | 121140 | Madagascar | 121011 | Sri Lanka | 117849 | Taiwan |
| 117840 | China | 121811 | Madagascar | 121094 | Sri Lanka | 117848 | Taiwan |
| 120912 | China | 121990 | Madagascar | 121153 | Sri Lanka | 117907 | Taiwan |
| 121042 | China | 121963 | Madagascar | 117525 | Sri Lanka | 121235 | Thailand |
| 120946 | China | 121142 | Madagascar | 121010 | Sri Lanka | 122179 | Thailand |
| 120861 | China | 121834 | Madagascar | 120893 | Sri Lanka | 121019 | Thailand |
| 117271 | China | 121717 | Madagascar | 121152 | Sri Lanka | 117561 | Thailand |
| 120972 | China | 121052 | Madagascar | 121131 | Sri Lanka | 121020 | Thailand |
| 117912 | China | 121839 | Madagascar | 121070 | Sri Lanka | 117587 | Thailand |
| 120947 | China | 121829 | Madagascar | 121098 | Sri Lanka | 122025 | Colombia |
| 117277 | China | 121830 | Madagascar | 121158 | Sri Lanka | 121147 | Colombia |
| 121001 | China | 121141 | Madagascar | 121059 | Sri Lanka | 117829 | Colombia |
| 121111 | China | 121812 | Madagascar | 121077 | Sri Lanka | 120863 | Colombia |
| 121163 | China | 121102 | India | 121127 | Sri Lanka | 122011 | Colombia |
| 121044 | China | 120867 | India | 121065 | Sri Lanka | 121016 | Myanmar |
| 121162 | China | 122206 | India | 121041 | Sri Lanka | 121080 | Myanmar |
| 122088 | Philippines | 120998 | India | 117567 | Sri Lanka | 121045 | Myanmar |
| 122099 | Philippines | 121774 | India | 120948 | Sri Lanka | 120904 | Myanmar |
| 122089 | Philippines | 121881 | India | 121083 | Sri Lanka | 120883 | Myanmar |
| 121835 | Philippines | 120882 | India | 120856 | Sri Lanka | 120949 | Brazil |
| 121591 | Philippines | 121130 | India | 120958 | Sri Lanka | 117463 | Brazil |
| 121753 | Philippines | 122181 | India | 117460 | Bangladesh | 117520 | Brazil |
| 120984 | Philippines | 121725 | India | 121034 | Bangladesh | 121053 | Brazil |
| 122272 | Philippines | 117915 | India | 117623 | Bangladesh | 121663 | Nepal |
| 121748 | Philippines | 117501 | India | 120879 | Bangladesh | 121225 | Nepal |
| 122091 | Philippines | 117500 | India | 121101 | Bangladesh | 121091 | Nepal |
| 122098 | Philippines | 121066 | India | 120913 | Bangladesh | 120959 | Nepal |
| 120983 | Philippines | 121118 | India | 121035 | Bangladesh | 121085 | Ecuador |
| 121087 | Philippines | 121064 | India | 121095 | Bangladesh | 120979 | Ecuador |
| 122236 | Philippines | 117564 | India | 117447 | Bangladesh | 121105 | Iran |
| 121760 | Philippines | 121172 | India | 121237 | Bangladesh | 120855 | Iran |
| 121023 | Philippines | 117521 | India | 121106 | Bangladesh | 117276 | Korea Rep |
| 122000 | Philippines | 117533 | India | 121146 | Bangladesh | 117531 | Korea Rep |
| 120981 | Philippines | 121139 | India | 120895 | Bangladesh | 120964 | Laos |
| 117757 | Philippines | 117563 | India | 121122 | Bangladesh | 120952 | Laos |
| 117758 | Philippines | 117691 | India | 120924 | Bangladesh | 120994 | Cuba |
| 121750 | Philippines | 121708 | India | 122298 | Indonesia | 120860 | Egypt |
| 117659 | Philippines | 122255 | India | 122029 | Indonesia | 120977 | Guatemala |
| 120982 | Philippines | 117454 | India | 120878 | Indonesia | 121169 | Guinea |
| 117268 | Philippines | 121689 | India | 121965 | Indonesia | 121062 | Kenya |
| 122094 | Philippines | 117598 | India | 117559 | Indonesia | 121420 | Malaysia |
| 121751 | Philippines | 120885 | Senegal | 117880 | Indonesia | 121051 | Mauritius |
| 120987 | Philippines | 122287 | Senegal | 117443 | Indonesia | 120939 | Upper volta |
| 122090 | Philippines | 121854 | Senegal | 121889 | Indonesia | 120916 | Venezuela |
| 121089 | Philippines | 121856 | Senegal | 122076 | Mali | 121165 | Zambia |
