# Supplementary material for: Genome-Wide Association Study of Grain Appearance and Milling Quality in a Worldwide Collection of Indica Rice Germplasm
Source: PLoS One. 2015 Dec 29;10(12):e0145577. doi: 10.1371/journal.pone.0145577 (PMC4694703; doi:10.1371/journal.pone.0145577)
Supplement: S2 Table — (DOCX) [file pone.0145577.s005.docx]

S2 Table. Distributions of markers on chromosomes.

| Chr | Marker No. | Size (Mb) | Average Distance (Kb) |
| --- | --- | --- | --- |
| Chr1 | 2,361 | 43.2 | 18.3 |
| Chr2 | 1,965 | 35.9 | 18.3 |
| Chr3 | 1,763 | 36.3 | 20.6 |
| Chr4 | 1,782 | 35.4 | 19.9 |
| Chr5 | 1,171 | 29.9 | 25.6 |
| Chr6 | 1,589 | 31.0 | 19.5 |
| Chr7 | 1,431 | 29.6 | 20.7 |
| Chr8 | 1,388 | 28.3 | 20.4 |
| Chr9 | 1,178 | 22.8 | 19.4 |
| Chr10 | 891 | 23.1 | 25.9 |
| Chr11 | 1,771 | 28.8 | 16.3 |
| Chr12 | 1,534 | 27.4 | 17.9 |
| Total | 18,824 | 371.7 | 20.2 |
