# Supplementary material for: Genome-Wide Association Study of Grain Appearance and Milling Quality in a Worldwide Collection of Indica Rice Germplasm
Source: PLoS One. 2015 Dec 29;10(12):e0145577. doi: 10.1371/journal.pone.0145577 (PMC4694703; doi:10.1371/journal.pone.0145577)
Supplement: S3 Table — (DOCX) [file pone.0145577.s006.docx]

**S3 Table. Basic characteristics of LD pattern in the whole panel and three subpopulations.**

| Population^a^ | Maximum r^2^ | Basal r^2^ | Decay distance (kb) | R^2b^ |
| --- | --- | --- | --- | --- |
| Whole | 0.65 | 0.11 | 150 | 0.87 |
| Q3-1 | 0.76 | 0.16 | 110 | 0.93 |
| Q3-2 | 0.88 | 0.16 | 240 | 0.88 |
| Q3-3 | 0.71 | 0.19 | 80 | 0.95 |

^a^ Subpopulations were inferred by STRUCTURE *k*=3.

^b^ R^2^ was the determination coefficient of the power law model.
