# Supplementary material for: Genome-Wide Association Study of Grain Appearance and Milling Quality in a Worldwide Collection of Indica Rice Germplasm
Source: PLoS One. 2015 Dec 29;10(12):e0145577. doi: 10.1371/journal.pone.0145577 (PMC4694703; doi:10.1371/journal.pone.0145577)
Supplement: S4 Table — (DOCX) [file pone.0145577.s007.docx]

**S4 Table.** **Comparsion of 16 association models based the mean squared difference (MSD) between the observed and expected p values for 40 trait (10)-environment (4) combinations.**

| Env_Trait | naive | Q3 | Q7 | C7 | Kp | Ks | Kv | Q3Kp | Q3Ks | Q3Kv | Q7Kp | Q7Ks | Q7Kv | C7Kp | C7Ks | C7Kv |
| --- | --- | --- | --- | --- | --- | --- | --- | --- | --- | --- | --- | --- | --- | --- | --- | --- |
| HZ_BRR | 0.0009 | 0.0012 | **0.0003** | 0.0007 | 0.0013 | 0.0016 | 0.0010 | 0.0009 | 0.0006 | 0.0010 | 0.0010 | 0.0019 | 0.0008 | 0.0009 | 0.0004 | 0.0008 |
| HZ_DEC | 0.0325 | 0.0219 | 0.0086 | 0.0157 | 0.0003 | 0.0003 | 0.0007 | 0.0003 | 0.0003 | 0.0008 | 0.0007 | **0.0002** | 0.0018 | **0.0002** | **0.0002** | 0.0010 |
| HZ_GL | 0.3075 | 0.0284 | 0.0144 | 0.0156 | 0.0046 | 0.0046 | 0.0022 | 0.0024 | 0.0038 | 0.0015 | 0.0016 | 0.0017 | **0.0012** | 0.0015 | 0.0014 | 0.0018 |
| HZ_GLWR | 0.8830 | 0.0702 | 0.0105 | 0.0214 | 0.0026 | 0.0026 | 0.0011 | 0.0013 | 0.0013 | 0.0005 | 0.0013 | 0.001 | 0.0008 | 0.0005 | 0.0012 | **0.0003** |
| HZ_GT | 0.1789 | 0.0106 | 0.0034 | 0.0076 | 0.0018 | 0.0018 | **0.0012** | 0.0019 | 0.0015 | 0.0031 | 0.0016 | **0.0012** | 0.0027 | 0.0021 | 0.0018 | 0.0028 |
| HZ_GW | 0.7308 | 0.0987 | 0.0100 | 0.0173 | 0.0012 | 0.0012 | 0.0005 | 0.0018 | 0.0018 | 0.0007 | 0.0003 | 0.0010 | 0.0004 | 0.0003 | 0.0004 | **0.0001** |
| HZ_HMRR | 0.0014 | **0.0011** | **0.0011** | **0.0011** | 0.0020 | 0.0063 | 0.0062 | 0.0013 | 0.0047 | 0.0063 | 0.0021 | 0.0041 | 0.0067 | 0.002 | 0.0052 | 0.0063 |
| HZ_TGW | 0.0053 | 0.0031 | 0.0037 | 0.0022 | 0.0018 | 0.0008 | 0.0047 | 0.0007 | 0.0007 | **0.0003** | 0.0012 | 0.0007 | 0.0048 | 0.0007 | 0.0007 | 0.0006 |
| HZ_MRR | 0.1554 | 0.1186 | 0.1321 | 0.1298 | 0.0094 | 0.0060 | 0.0111 | 0.0076 | 0.0073 | 0.0099 | 0.0052 | 0.0117 | 0.0081 | 0.0060 | 0.0121 | **0.0027** |
| HZ_PGWC | 0.0259 | 0.0286 | 0.0044 | 0.0100 | **0.0001** | **0.0001** | 0.0002 | 0.0002 | 0.0002 | **0.0001** | 0.0004 | 0.0002 | **0.0001** | 0.0002 | 0.0003 | 0.0003 |
| JZ_BRR | 0.0871 | 0.0888 | 0.0904 | 0.1131 | 0.0004 | 0.0002 | 0.0788 | 0.0004 | **0.0001** | 0.0789 | 0.0003 | **0.0001** | 0.0764 | 0.0005 | 0.0003 | 0.0900 |
| JZ_DEC | 0.9647 | 0.0050 | 0.0019 | **0.0007** | 0.0022 | 0.0019 | 0.0031 | 0.0019 | 0.0009 | 0.0044 | 0.0013 | 0.0008 | 0.0035 | 0.0018 | 0.0011 | 0.0049 |
| JZ_GL | 0.7024 | 0.0056 | 0.0053 | 0.007 | 0.0013 | 0.0013 | **0.0002** | **0.0002** | **0.0002** | 0.0005 | 0.0003 | 0.0004 | **0.0002** | 0.0004 | 0.0004 | 0.0005 |
| JZ_GLWR | 1.3391 | 0.0596 | 0.0058 | 0.0168 | 0.0037 | 0.0037 | 0.0007 | 0.0020 | 0.0020 | **0.0002** | 0.0005 | 0.0005 | 0.0016 | 0.0008 | 0.0010 | 0.0012 |
| JZ_GT | 0.3433 | 0.0036 | 0.0009 | 0.0007 | 0.0017 | 0.0017 | 0.0056 | 0.0009 | 0.0010 | 0.0066 | **0.0001** | **0.0001** | 0.0062 | **0.0001** | 0.0003 | 0.0052 |
| JZ_GW | 1.2121 | 0.1174 | 0.0072 | 0.0133 | 0.0047 | 0.0047 | **0.0007** | 0.0042 | 0.0042 | 0.0028 | 0.0015 | 0.0033 | 0.0012 | 0.0014 | 0.0017 | 0.0029 |
| JZ_HMRR | 0.0622 | 0.0218 | 0.0174 | 0.015 | 0.0007 | 0.0005 | 0.0031 | 0.0007 | **0.0004** | 0.0126 | 0.0015 | 0.0008 | 0.0068 | 0.0011 | 0.0007 | 0.0041 |
| JZ_TGW | 0.0008 | 0.0009 | 0.0004 | 0.0016 | 0.0015 | 0.0011 | 0.0051 | 0.0009 | 0.0005 | 0.0047 | 0.0009 | 0.0004 | 0.0042 | 0.0007 | **0.0002** | 0.0034 |
| JZ_MRR | 0.0232 | 0.0181 | 0.0270 | 0.0269 | 0.0016 | 0.0014 | 0.0093 | 0.0016 | 0.0013 | 0.0057 | 0.0017 | 0.0016 | 0.0071 | 0.0015 | **0.0012** | 0.0116 |
| JZ_PGWC | 0.7402 | 0.0185 | 0.0139 | 0.0213 | 0.0010 | 0.0007 | 0.0276 | 0.0025 | **0.0002** | 0.0222 | 0.0020 | 0.0026 | 0.0194 | 0.0013 | 0.0011 | 0.0229 |
| SY_BRR | **0.0006** | 0.0055 | 0.0079 | 0.0057 | 0.0066 | 0.0018 | 0.0027 | 0.0154 | 0.0073 | 0.0191 | 0.0189 | 0.0106 | 0.0225 | 0.0154 | 0.0085 | 0.0502 |
| SY_DEC | 0.1313 | 0.0247 | 0.0196 | 0.0296 | 0.0007 | 0.0007 | 0.0124 | 0.0007 | 0.0008 | 0.0103 | **0.0001** | **0.0001** | 0.0094 | 0.0008 | 0.0008 | 0.0117 |
| SY_GL | 0.6315 | 0.0646 | 0.0365 | 0.0617 | 0.0090 | 0.0090 | 0.0140 | 0.0061 | 0.0061 | 0.0112 | 0.0040 | 0.0040 | **0.0023** | 0.0060 | 0.0060 | 0.0148 |
| SY_GLWR | 1.3241 | 0.1881 | 0.0242 | 0.1120 | 0.0074 | 0.0074 | 0.0061 | 0.0043 | 0.0043 | 0.0014 | 0.0061 | 0.0079 | **0.0010** | 0.0039 | 0.0039 | 0.0069 |
| SY_GT | 0.0746 | 0.0097 | 0.0050 | 0.0062 | 0.0008 | 0.0009 | 0.0051 | 0.0006 | 0.0007 | 0.0046 | 0.0002 | **0.0001** | 0.0057 | 0.0006 | 0.0005 | 0.0040 |
| SY_GW | 0.9470 | 0.1487 | 0.0033 | 0.0402 | 0.0017 | 0.0017 | **0.0004** | 0.0014 | 0.0014 | 0.0004 | 0.0028 | 0.0252 | 0.0009 | 0.0021 | 0.0011 | 0.0007 |
| SY_HMRR | 0.0084 | 0.0025 | 0.0028 | 0.0017 | 0.0006 | 0.0006 | 0.0157 | **0.0005** | 0.001 | 0.0115 | 0.0013 | 0.0024 | 0.0009 | 0.0009 | 0.0011 | 0.0125 |
| SY_TGW | 0.0075 | 0.0072 | 0.0072 | 0.0072 | 0.0017 | 0.0014 | 0.0023 | 0.0011 | 0.0011 | **0.0007** | 0.0010 | 0.0010 | 0.0018 | 0.0016 | 0.0013 | 0.0019 |
| SY_MRR | 0.0037 | 0.0019 | **0.0013** | 0.0023 | 0.0108 | 0.022 | 0.0081 | 0.0074 | 0.0173 | 0.0059 | 0.006 | 0.0167 | 0.0324 | 0.0081 | 0.0196 | 0.0357 |
| SY_PGWC | 0.2034 | 0.0486 | 0.0514 | 0.0712 | 0.0004 | 0.0004 | 0.0122 | 0.0003 | 0.0004 | 0.0107 | **0.0002** | **0.0002** | 0.0100 | **0.0002** | **0.0002** | 0.0121 |
| SZ_BRR | 0.1388 | 0.0092 | 0.0033 | 0.0009 | 0.0004 | 0.0006 | 0.0046 | 0.0004 | 0.0034 | 0.0008 | 0.0006 | 0.0007 | 0.0006 | 0.0004 | **0.0001** | 0.0006 |
| SZ_DEC | 0.0443 | 0.0090 | 0.0063 | 0.0093 | 0.0015 | 0.002 | 0.0012 | 0.0009 | 0.0015 | 0.0008 | 0.0004 | **0.0002** | 0.0008 | 0.0015 | 0.0021 | 0.001 |
| SZ_GL | 0.3291 | 0.0392 | 0.0135 | 0.0209 | 0.0041 | 0.0041 | 0.0033 | 0.0022 | 0.0022 | 0.0028 | 0.0018 | 0.0014 | 0.0015 | **0.0012** | **0.0012** | 0.0026 |
| SZ_GLWR | 1.1181 | 0.1524 | 0.0116 | 0.0570 | 0.0039 | 0.0039 | 0.0044 | 0.0021 | 0.0021 | 0.0028 | 0.0027 | 0.0021 | 0.0038 | 0.0019 | **0.0009** | 0.0020 |
| SZ_GT | 0.2610 | 0.0294 | 0.0063 | 0.0109 | 0.004 | 0.0040 | 0.0042 | 0.0041 | 0.0038 | 0.0042 | 0.0035 | 0.0035 | 0.0039 | **0.0034** | **0.0034** | 0.0039 |
| SZ_GW | 1.0685 | 0.1834 | 0.0150 | 0.0531 | 0.0031 | 0.0031 | 0.0054 | 0.0022 | 0.0022 | 0.0025 | 0.003 | 0.0043 | 0.0022 | **0.0017** | 0.0023 | **0.0017** |
| SZ_HMRR | 0.0027 | 0.0018 | 0.0013 | 0.0014 | **0.0006** | 0.0007 | 0.0215 | 0.0022 | 0.0086 | 0.0196 | 0.0015 | 0.0018 | 0.0155 | 0.0017 | 0.0078 | 0.0103 |
| SZ_TGW | 0.0633 | 0.0149 | 0.0188 | 0.0207 | 0.0042 | 0.0018 | 0.0006 | 0.0017 | 0.0016 | **0.0004** | 0.0021 | 0.0015 | 0.0005 | 0.0033 | 0.0019 | 0.0005 |
| SZ_MRR | 0.5278 | 0.5523 | 0.5593 | 0.3175 | 0.0044 | 0.0639 | 0.1133 | 0.0041 | 0.0681 | 0.1177 | 0.0022 | 0.0691 | 0.113 | **0.0022** | 0.1569 | 0.0544 |
| SZ_PGWC | 0.0683 | 0.0257 | 0.0187 | 0.0278 | 0.0045 | 0.0045 | 0.0036 | 0.0030 | 0.0030 | 0.0028 | 0.0023 | 0.0023 | **0.0021** | 0.0035 | 0.0035 | 0.0031 |
| Average | 0.3688 | 0.0560 | 0.0293 | 0.0324 | 0.0029 | 0.0044 | 0.0101 | 0.0024 | 0.0042 | 0.0098 | 0.0022 | 0.0047 | 0.0096 | 0.0021 | 0.0064 | 0.0099 |

Values in bold were the lowest for the corresponding trait-environment combination.
